# An Efficient Certificateless Anonymous Signcryption Scheme for WBAN

**DOI:** 10.3390/s24154899

**Published:** 2024-07-28

**Authors:** Weifeng Long, Lunzhi Deng, Jiwen Zeng, Yan Gao, Tianxiu Lu

**Affiliations:** 1 School of Mathematical Sciences, Xiamen University, Xiamen 361005, China; jwzeng@xmu.edu.cn; 2 Guizhou Provincial Specialized Key Laboratory of Information Security Technology in Higher Education Institutions, School of Mathematical Sciences, Guizhou Normal University, Gui’an New District, Guiyang 550025, China; denglunzhi@163.com (L.D.); 19010060164@gznu.edu.cn (Y.G.); 232200061298@gznu.edu.cn (T.L.)

**Keywords:** signcryption, certificateless cryptography, Wireless Body Area Networks (WBANs), standard model, efficient, security

## Abstract

A Wireless Body Area Network (WBAN), introduced into the healthcare sector to improve patient care and enhance the efficiency of medical services, also brings the risk of the leakage of patients’ privacy. Therefore, maintaining the communication security of patients’ data has never been more important. However, WBAN faces issues such as open medium channels, resource constraints, and lack of infrastructure, which makes the task of designing a secure and economical communication scheme suitable for WBAN particularly challenging. Signcryption has garnered attention as a solution suitable for resource-constrained devices, offering a combination of authentication and confidentiality with low computational demands. Although the advantages offered by existing certificateless signcryption schemes are notable, most of them only have proven security within the random oracle model (ROM), lack public ciphertext authenticity, and have high computational overheads. To overcome these issues, we propose a certificateless anonymous signcryption (CL-ASC) scheme suitable for WBAN, featuring anonymity of the signcrypter, public verifiability, and public ciphertext authenticity. We prove its security in the standard model, including indistinguishability, unforgeability, anonymity of the signcrypter, and identity identifiability, and demonstrate its superiority over relevant schemes in terms of security, computational overheads, and storage costs.

## 1. Introduction

The latest data by the World Health Organization (WHO) reveals that the average global life expectancy has reached 73.4 years. Furthermore, demographic projections estimate that by 2050, the number of individuals aged 60 and above will surge to 2.1 billion. This demographic shift towards an older population is exacerbating the shortage of medical resources, making healthcare for the elderly a critical issue for nations worldwide. The escalating costs of healthcare have driven medical systems to embrace new technologies to enhance current practices. To capitalize on the benefits of wireless technology in the realms of telemedicine and mobile health, a novel type of wireless network has emerged: the Wireless Body Area Network (WBAN) [1]. The WBAN is a specialized sensor network that facilitates the exchange of vital health information between patients and healthcare providers via the internet.

**Figure 1:**
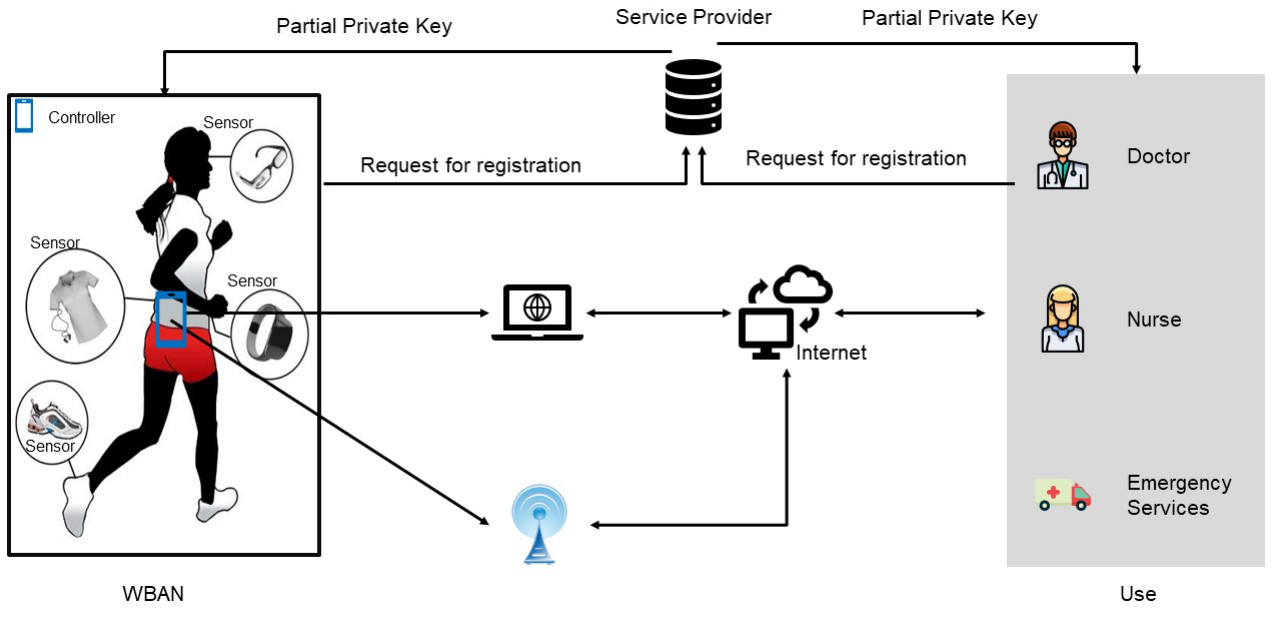
WBAN Framework.

A standard WBAN encompasses an array of either implantable [2,3] or wearable sensor nodes and control units [4]. The role of these sensor nodes is to diligently monitor the critical physiological parameters of individuals, covering a range of critical health indicators such as blood pressure, oxygen saturation levels, respiratory rate, heart rate, skin temperature, and various other essential signs of life. In addition to these vital signs, they also measure environmental factors, such as ambient temperature, humidity levels, and light intensities. The sensor nodes engage in communication with a central controller, which acts as a conduit for relaying the aggregated health data to medical personnel and servers within the network. The WBAN framework is shown in Figure 1. The implementation of WBAN has significantly enhanced the efficiency of healthcare delivery, as it reduces the frequency with which patients need to visit hospitals. Furthermore, the system is capable of facilitating clinical diagnoses and providing some emergency medical responses. Given the significant role that WBAN will play in the healthcare system, it is projected that the WBAN market will exceed 19 trillion US dollars in the next few years [5]. It is expected that there will be 100 billion Internet of Things (IoT) devices in operation globally by the year 2025, with an expected economic impact that will exceed 11 trillion US dollars [6].

Garnering enormous economic interest, WBAN may be confronted with the risks of data misuse and infringement of user privacy. Although various countries are continuously improving their regulatory systems, their strategies focus on effectiveness and security. For example, the EU’s General Data Protection Regulation (GDPR), which came into effect in May 2018, granted privacy regulatory authorities the right to impose fines or file lawsuits against individual companies. It drives societal attention to privacy and security. Whitefield Diffie and Susan Landau concluded that we can best protect our communications through encryption in their book Privacy on the Line. Cryptography has long been a tool for securing communications and protecting privacy. The fundamental goal of cryptography is to achieve secure communication, and it has been observed that the privacy of ordinary people may be infringed upon in communications, which has led to questions being raised about the field of cryptography. The cryptography community has begun to focus on the social impact of its work. For instance, the Association for Computing Machinery (ACM) upholds detailed codes of ethics and professional conduct, including directives on honesty, privacy, and societal contribution [7]. The American Mathematical Society (AMS) and Mathematical Association of America (MAA) provide more generalized guidance on ethical conduct: The MAA requires Directors, Officers, Members, those compensated by the MAA and those donating their time, and all employees, to observe high standards of business and personal ethics in the conduct of their duties and responsibilities [8]. When mathematical work may affect the public health, safety or general welfare, it is the responsibility of mathematicians to disclose the implications of their work to their employers and to the public, if necessary [9]. Yet, the International Association for Cryptologic Research (IACR), despite its focus on cryptography, lacks a comprehensive ethical statement. Phillip Rogaway [10] emphasized the ethical responsibilities of cryptography work, not only to focus on technical and mathematical challenges but also to recognize the impact of their work in society, and to be driven by ethics to make more meaningful contributions to society. Designing cryptography schemes requires a reflective approach that navigates complex ethical terrains, considering how cryptography tools and techniques affect social norms and values, and is capable of both protecting individual privacy and enabling surveillance.

The core element of ensuring the security of WBAN systems lies in the establishment of an efficient security framework. Within this framework, the two major security challenges of authentication and confidentiality are particularly crucial and require urgent solutions. In response to these challenges, encryption technology and digital signatures have been widely adopted as effective means to enhance security and verification mechanisms. In practice, when both encryption and signature functions are required concurrently, a common approach is to prioritize signature processing followed by encryption, in order to ensure the integrity and confidentiality of information. However, given the stringent constraints of low-power sensor devices in WBANs, such as limited onboard energy and central processing unit (CPU) processing capabilities, executing complex encryption programs appears impractical. To overcome this technical barrier, an innovative “signcryption” technology [11] has emerged, which ingeniously combines the functions of signing and encryption. This not only simplifies the operational process but also adapts to resource-constrained environments. Most importantly, compared to the traditional method of signing first and then encrypting, signcryption technology exhibits greater applicability in resource-constrained application scenarios such as WBANs due to its higher cost-effectiveness.

Currently, in response to the security challenges faced by WBANs, scholars have conducted extensive research from multiple angles and designed a series of signcryption schemes to tackle the security challenges faced by WBANs [12,13,14,15,16]. The core of these schemes lies in the establishment of three major cryptographic systems: the Public Key Cryptography (PKC), Identity-based Public Key Cryptography (ID-PKC), and Certificateless Public Key Cryptography (CL-PKC). During the process of system deployment, PKC often confronts intricate challenges related to certificate management. While ID-PKC can effectively bypass the difficulties of certificate management encountered in PKC, its drawback lies in the necessity of implementing a key escrow mechanism. Although a lightweight ID-PKC is highly suitable for resource-constrained WBANs, the security is compromised when the Private Key Generator (PKG) is compromised, as the PKG learns the private keys of all users. In other words, the PKG can decrypt ciphertexts in Identity-Based Encryption (IBE) schemes and can forge signatures for messages in Identity-Based Signature (IBS) schemes. Therefore, ID-PKC is only suitable for small-scale networks like WBANs, rather than large-scale networks such as the Internet. In this context, where the communication between Internet users and WBANs is being considered, CL-PKC emerges as an ideal choice compared to ID-PKC.

However, WBAN utilizes public communication channels, making the transmitted data highly vulnerable to eavesdropping, interception, replay, forgery, and tampering by adversaries. Therefore, it is very important to design an efficient and secure CLSC scheme to realize secure communication in WBAN. In order to achieve security, we must overcome a series of technical challenges [17,18,19]. The scope of these challenges covers a wide range of issues, including confidentiality, integrity, authentication, non-repudiation [5], anonymity, public verifiability, and public ciphertext authenticity. To tackle the aforementioned challenges, we present a certificateless anonymous signcryption (CL-ASC) scheme specifically for WBAN. Under the standard model, we have demonstrated that the scheme satisfies the requirements of anonymity of the signcrypter, and identity identifiability.

### 1.1. Related Work

The following related work can be focused on from two aspects: firstly, research regarding the CLSC Scheme itself; secondly, the application and exploration of the CLSC scheme within WBANs.

To eliminate key escrow in ID-PKC and simplify the certificate management in traditional PKC, Al-Riyami and Paterson [20] introduced the concept of CL-PKC. In CL-PKC, a user’s complete private key comprises two parts: one is a partial private key generated by KGC, and the other is a secret value generated by the user themselves. Additionally, public keys do not require certificates. Therefore, the certificateless public key cryptosystem boasts significant advantages and has garnered widespread attention since its inception [21,22,23,24]. In 2008, Barbosa and Farshim [23] combined the certificateless public key system with signcryption to introduce the Certificateless Signcryption (CLSC) scheme, while also defining the formal security concepts of CLSC schemes. The certificateless signcryption has the advantages of both the certificateless public key cryptographic system and signcryption. Building on this foundational work, numerous CLSC schemes have been proposed [25,26,27,28,29,30,31,32], but most of them have been proven secure in the ROM. It is well known that proofs in the ROM serve only as heuristic evidence and do not necessarily imply security in practical implementations [33]. Therefore, it is imperative to consider how to construct provably secure schemes without relying on random oracles. In 2010, Liu et al. [24] first proposed a certificateless signcryption scheme in the standard model; unfortunately, this model is insecure in the face of a malicious but passive Key Generation Center (KGC) and a public key substitution attack [34,35,36]. Subsequently, Jin et al. [37] adopted a new method to optimize and improve Liu’s scheme and proved that their improved scheme is secure in the standard model. However, Xiong [38] demonstrated that Jin’s scheme is not resistant to chosen ciphertext attacks and is vulnerable to malicious but passive KGC attacks. In 2017, Luo et al. [28] constructed a CLSC scheme and claimed to achieve unforgeability against adaptive chosen message attacks and ciphertext indistinguishability against adaptive chosen ciphertext attacks in the standard model. However, Yuan [39] pointed out that the scheme [28] failed to fulfil its purported security claims. Subsequently, Rastegari et al. [40] discovered a critical flaw in the scheme and proposed a revised CLSC scheme, but Lin [41] analyzed it and concluded that the scheme [40] was insecure. Therefore, how to propose a secure certificateless signcryption scheme under the standard model remains an open question.

There are two types of adversaries in certificateless cryptosystems. The Type I adversary, $A_{1}$, mimics an “external” adversary who does not know the master secret key but can replace anyone’s public key. The Type II adversary, $A_{2}$, mimics an “internal” adversary who knows the master secret key but cannot replace anyone’s public key. It should be noted that $A_{2}$ only encompasses the “honest-but-curious” KGC, but a malicious and passive KGC may attempt to decrypt ciphertexts or forge signatures by embedding additional trapdoors in the public parameters [29]. Therefore, a stronger security model is needed to capture the operations of a malicious yet passive KGC. In 2007, Au et al. [42] introduced the concept of a malicious yet passive KGC as a Type II adversary. This type of attacker is malicious during the initial setup phase of the system, thereby allowing the Type II adversary to generate all public parameters and the master secret key. For adversary $A_{2}$, a malicious yet passive $A_{2}$ attack is more realistic and powerful than an honest-but-curious $A_{2}$ attack. To resist attacks by a malicious but passive KGC and public key substitution attacks, we consider the malicious but passive KGC as a Type II adversary $A_{2}$ in our security model and grant $A_{2}$ the ability to replace public keys.

In 2016, Li et al. [43] debuted a CLSC scheme aimed at WBAN access control, claiming it met various security criteria, such as authentication, confidentiality, and non-repudiation, indicating its broad applicability. Unfortunately, the scheme was still vulnerable to replay attacks and lacked public verifiability [44]. In 2018, Li et al. [45] proposed a new CLSC scheme within an economical and anonymous access control mechanism for WBAN, claiming it encompassed security features like anonymity, confidentiality, authentication, integrity, and non-repudiation. However it is noteworthy that their security proofs were conducted in the ROM, and it lacked consideration for public verifiability and publicly ciphertext authenticity. In the same year, Lu et al. [46] developed a traceable threshold attribute signature scheme, aimed at providing better security for mobile healthcare social networks (MHSN). The article claims that the scheme has correctness, unforgeability, traceability, and privacy. However, the security proof of the scheme is also implemented in the ROM and lacks public verifiability and public ciphertext authenticity. In 2018, Liu [47] proposed a lightweight CLSC scheme based on RSA, and designed a lightweight and efficient WBAN data access control scheme. The article claims that the scheme can meet more security requirements in WBAN. However, the scheme’s security is only proven in the ROM. The existing certificateless signcryption-based data access control schemes have the following two weaknesses: (1) most of the security proofs are implemented in ROM. (2) most of the schemes lack anonymity, public verifiability, and publicly ciphertext authenticity. Subsequently, the use of our proposed CLSC scheme to design an efficient and secure WBAN data access control scheme can be considered.

### 1.2. Motivations and Contributions

Wireless Body Area Networks (WBANs) play a significant role in monitoring health information and creating efficient healthcare systems. The task of designing a secure and economical communication scheme suitable for WBANs is made particularly challenging due to the inherent characteristics of WBANs, such as the open medium channel and the limited resources of sensor nodes. Signcryption is an encryption technology that can simultaneously achieve the functions of public key encryption and digital signatures, which can authenticate users and protect query messages at the same time. It can achieve confidentiality, authentication, integrity, and non-repudiation at a low cost, which is suitable for WBANs. The CLSC schemes proposed in recent years have the following weakness:

- The security proofs of most schemes are implemented in ROM. However, the CLSC schemes with provable security in ROM may have vulnerabilities in practical applications.
- A Type II adversary in the security models of most schemes is considered a “honest but curious” KGC, but in reality, this may be a “malicious but passive” KGC.
- The schemes lack public verifiability and public ciphertext authenticity. This leads to the receiver having to decrypt the ciphertext first and then verify its validity. If the ciphertext is invalid, the decryption work will be wasted.
- Most schemes do not have anonymity of the signcrypter. This is not conducive to protecting the privacy of the sender.
- High computational cost. In order to complete a signcryption–unsigncryption algorithm, the scheme requires multiple pairing operations, which is not suitable for low-power devices.

Therefore, the purpose of this paper was to introduce a scheme that is both efficient and secure, addressing the aforementioned concerns. The contributions of this paper are as follows:

- We introduce a CL-ASC scheme which is suitable for WBAN, with anonymity of the signcrypter, public verifiability, public ciphertext authenticity, and identifiable identity. There are very few CL-ASC schemes that have all these special features. Compared to other schemes, our scheme has very powerful functions and shows some degree of innovation.
- We provided a stronger security model for the CL-ASC scheme. Our security model considers a malicious but passive KGC as a Type II adversary, which can generate all public parameters and the master secret key during the initial system setup stage, and is endowed with stronger capabilities. In addition, both Type I and Type II adversaries can directly compute hash functions to obtain results. This significantly enhances the capabilities of the adversaries, making the scheme more secure and more aligned with real-world scenarios.
- We demonstrate that our scheme possesses indistinguishability, unforgeability, anonymity of the signcrypter, and identity identifiability in the standard model.
- Compared to the related schemes, our scheme offers superior security performance, along with reduced computational overheads and storage costs, and offers better security, making it more suitable for WBAN.

### 1.3. Organization

The subsequent sections are structured as follows: in Section 2, we introduce the fundamental concepts; Section 3 elaborates on the system’s architecture; Section 4 specifies the security framework; the proposed scheme is thoroughly described in Section 5; and its security analysis is presented in Section 6. A comparative analysis of performance is presented in Section 7; and Section 8 summarizes the conclusions of our study.

## 2. Preliminaries

The structure is distinguished by the presence of an additive cyclic group $G_{1}$ and a multiplicative cyclic group $G_{2}$, each possessing an order of *q*, with *q* being a prime number. A bilinear map, denoted by $e:G_{1}\times G_{1}\to G_{2}$, is defined by the following properties:

Non-degeneracy: There exist $P,Q\in G_{1}$ such that $e\left(P,Q\right)\neq 1_{G_{1}}$.

Computability: An efficient computational method exists for determining e(P,Q) for any given *P* and *Q* from their respective groups.

Bilinearity: For every pair of elements $P,Q\in G_{1}$ and integers $a,b\in Z_{q}$, the map satisfies $e\left(aP,bQ\right)=e{\left(P,Q\right)}^{ab}$.

This mapping is referred to as bilinear, as described in [48].

The mathematical problems and assumptions about bilinear mapping used in this paper are as follows:

**Definition 1.**  Decisional Diffie–Hellman Problem (DDHP): When presented with elements P,aP,bP,X∈G, verify if X is indeed abP. Here, $P\in G_{1}$ and $a,b\in Z_{q}^{*}$.

**Definition 2.**  *Decisional Diffie–Hellman Assumption (DDHA): Under the DDHA, it is assumed that the likelihood of any algorithm capable of operating within polynomial time successfully resolving the DDHP is minimal.*

**Definition 3.**  Computational Attack Algorithm Problem(CAAP) [31]: Given a tuple (P,aP) for $a\in Z_{q}^{*},P\in G_{1}$, output a tuple $\left(c,\frac{1}{a+c}P\right)$.

**Definition 4.**  *Computational Attack Algorithm Assumption (CAAA): Under the CAAA, it is assumed that the likelihood of any algorithm capable of running in polynomial time successfully resolving the CAAP is minimal.*

## 3. System Model

The fundamental security prerequisites for the deployment of a signcryption scheme within WBAN are outlined as follows:

(1) Confidentiality: this means that any unauthorized party, other than the authorized individual or entity, cannot access the data content. Even if an unauthorized user obtains the encrypted data, they cannot decipher the true content of the data.

(2) Authentication: this refers to the authentication of data sources or entities.

(3) Integrity: guaranteeing the integrity of data transmitted in the network, preventing illegal entities from tampering with or deleting query messages.

(4) Non-repudiation: ensuring that the sender of data cannot deny previous commitments or actions.

(5) Unforgeability: if the attacker can forge the patient’s signature, the doctor will face obstacles in diagnosis and treatment, which may endanger the patient’s life. Therefore, we need the signcryption scheme to be unforgeable under the adaptive chosen message attack.

(6) Anonymity of the signcryptor: in order to protect user privacy, no other entity apart from KGC can indeed ascertain the true identity of the signcryptor.

(7) Identity identifiability: while ensuring user privacy, KGC is capable of verifying and tracing the identity of the signcryptor to ensure the security and credibility of data transmission and usage. Meanwhile, other unauthorized entities are prevented from accessing this sensitive information.

(8) Public verifiability [18]: a third party is registered to affirm the legitimacy of the encrypted message, independent of the access to the sender’s private key.

(9) Public ciphertext authenticity [18]: a third party can confirm the authenticity of the ciphertext without the need for decryption, allowing the receiver to discard invalid ciphertexts in advance, saving energy consumption and computation time, which is crucial for small devices.

**Figure 2:**
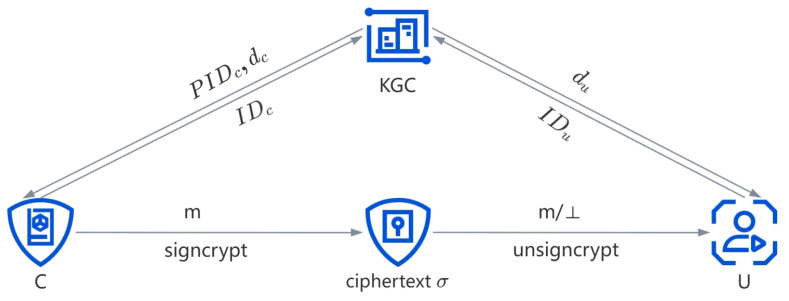
Schematic of system model.

The system model proposed in this paper is shown in Figure 2, which includes three entities: KGC, sender C, receiver U.

- KGC: Responsible for setting system parameters and publishing them publicly. Additionally, it is also responsible for generating pseudo-identities for sender C and generating partial private keys for both sender C and receiver U.
- C: Uses their own private key to perform signcryption on the data *m*, generates the ciphertext σ of *m*, and sends the ciphertext σ to B.
- U: Decrypts the ciphertext Upon receiving it, using their own private key to obtain the data *m*.

The CL-ASC scheme consists of eight distinct algorithms, each of which is delineated as follows:

- Setup(μ): Input parameter μ for security; KGC generates the system parameters params and master secret key msk. Then KGC has the public params, and secretly holds msk.
- PIDG($ID_{c}$, params): Input the real identity $ID_{c}$ of C; KGC generates a pseudo-identity $PID_{c}$ of C, and sends it to C.
- PPKG($ID_{u}$/$PID_{c}$, params, msk): Upon receiving the identity $ID_{u}$ of U (or the pseudo-identity $PID_{c}$ of C), KGC generates the partial private key $d_{u}$ (or $d_{c}$) of U (of C) and transmits it securely to U (or C).
- SVS($ID_{u}$/$PID_{c}$, params): U (or C) sets $x_{u}$ (or $x_{c}$) as its secret value.
- FSKS($ID_{u}$/$PID_{c}$, params, $x_{u}$/$x_{c}$, $d_{u}$/$d_{c}$): U (or C) sets its full private key $SK_{u}$ (or $SK_{c}$) as $SK_{u}=\left(x_{u},d_{u}\right)$ (or $SK_{c}=\left(x_{c},d_{c}\right)$).
- UPKG($ID_{u}$/$PID_{c}$, params, $X_{u}$/$X_{c}$, $R_{u}$/$R_{c}$): U (or C) sets its public key as $PK_{u}=\left(X_{u},R_{u}\right)$ (or $PK_{c}=\left(X_{c},R_{c}\right)$).
- Signcrypt$\left(m,ID_{u},PK_{u},PID_{c},SK_{c},params\right)$: Takes params, message *m*, U’s identity $ID_{u}$, U’s public key $PK_{u}$, C’s pseudo-identity $PID_{c}$ and C’s full private key $SK_{c}$ as input; C returns the ciphertext σ and transmits it to U.
- Unsigncrypt$\left(\sigma ,ID_{u},SK_{u},PID_{c},PK_{c},params\right)$: Takes params, ciphertext σ, U’s identity $ID_{u}$, U’s full private key $SK_{u}$, C’s pseudo-identity $PID_{c}$ and C’s public key $PK_{c}$ as input; U returns the corresponding plaintext *m* or ⊥.

## 4. Security Model

Based on the security models proposed by Barbosa et al. [23], Zhou et al. [30] and Deng [49], we present a security model for CL-ASC, and give the following explanations. Against a Type I adversary $A_{1}$, we have adopted the original security model proposed by Barbosa and Farshim, based on its notable advantage over another security model, namely that in the latter, in the public key replacement oracle, $A_{1}$ needs to provide the corresponding secret value when replacing the user’s public key. Barbosa and Farshim’s model demonstrates greater defensive capabilities. For a Type II adversary $A_{2}$, our security model takes into account a malicious yet passive KGC as $A_{2}$. At this time, $A_{2}$ can generate all public parameters and the master key during the initialization phase of the system, given that in practical scenarios, Type 2 adversaries also possess the capability to perform public key replacement attacks. Therefore, we allow $A_{2}$ to execute the public key replacement query in our security model, ensuring that the security model effectively defends against such threats. Furthermore, both Type I and Type II adversaries are capable of directly computing hash functions to obtain results.

Based on the above analysis, against indistinguishability under an adaptive chosen ciphertext attack and unforgeability under an adaptive chosen message attack, we present two types of adversaries.

$A_{1}$: $A_{1}$ is a dishonest user who can replace the public key of any entity with a value of their own choice, but they do not have access to the secret master key.

$A_{2}$: $A_{2}$ represents a malicious but passive KGC that generates all public parameters and master secret key and can perform public key replacement.

In addition, We introduce a super adversary, A, specifically targeting the anonymity of the signcryptor. A is a super adversary who possesses the capabilities of both $A_{1}$ and $A_{2}$, meaning that A is endowed with the capacity to replace users’ public keys and also has access to the master secret key, and can perform secret value queries. However, A is unable to access the list FI and cannot query the pseudo-identity of the target user.

**Definition 5.**  *If the adversary cannot win the following game with a non-negligible probability in any polynomial time, then the security property of the CLSC scheme is said to satisfy indistinguishability under an adaptive chosen ciphertext attack ($IND-CCA_{2}$).*

**Game 1**: The game between the adversary $A_{1}$ and the challenger B unfolds as follows:

- **Initialization phase**: B obtains msk and params by executing the setup algorithm, then sends params to $A_{1}$ and maintains the secrecy of msk.
- **Query phase**: For C, the queries $Q_{pid}\left(ID_{c}\right)$ and $Q_{upk}\left(PID_{c}\right)$ are executed before any other queries. For U, the query $Q_{upk}\left(ID_{u}\right)$ should be executed before any other queries. $A_{1}$ performs the following types of queries:
  $Q_{pid}\left(ID_{c}\right)$: $A_{1}$ sends a user identity $ID_{c}$ to B, B returns the pseudo-identity $PID_{c}$ to $A_{1}$.
  $Q_{upk}\left(PID_{c}/ID_{u}\right)$: $A_{1}$ sends a user identity $PID_{c}/ID_{u}$ to B, B returns the corresponding public key $PK_{c}/PK_{u}$ to $A_{1}$.
  $R_{upk}\left(PID_{c},PK_{c}^{\prime }\right)/\left(ID_{u},PK_{u}^{\prime }\right)$: $A_{1}$ sends a tuple $\left(PID_{c},PK_{c}^{\prime }\right)/\left(ID_{u},PK_{u}^{\prime }\right)$ to B, B replaces $PK_{c}/PK_{u}$ with $PK_{c}^{\prime }/PK_{u}^{\prime }$.
  $Q_{ppk}\left(PID_{c}/ID_{u}\right)$: $A_{1}$ sends a user identity $PID_{c}/ID_{u}$ to B, B returns the partial private key $d_{c}/d_{u}$ to $A_{1}$. When $R_{c}/R_{u}$ is replaced, $A_{1}$ cannot perform this query. The reason for imposing this restriction is that it is unreasonable to expect the challenger to provide a partial private key for users who do not know a partial private key.
  $Q_{sv}\left(PID_{c}/ID_{u}\right)$: $A_{1}$ sends a user identity $PID_{c}/ID_{u}$ to B, B returns the secret value $x_{c}/x_{u}$ to $A_{1}$. When $X_{c}/X_{u}$ is replaced, $A_{1}$ cannot perform this query. The reason for imposing this restriction is that it is unreasonable to expect the challenger to provide a secret value for users who do not know a secret value.
  $Q_{sc}\left(m,ID_{u},PK_{u},PID_{c},PK_{c}\right)$: $A_{1}$ sends tuple $\left(m,ID_{u},PK_{u},PID_{c},PK_{c}\right)$ to B, where *m* is the plaintext intended for signcryption, $ID_{u}$ is the identity of U, $PK_{u}$ is the public key of U whose identity is $ID_{u}$, $PID_{c}$ is the identity of C and $PK_{c}$ is the public key of C whose identity is $PID_{c}$. B first executes the PPKG algorithm, SVS algorithm and FSKS algorithm using identity $PID_{c}$ to obtain $SK_{c}$, and then executes the signcrypt algorithm using the tuple $\left(\sigma ,ID_{u},PK_{u},PID_{c},SK_{c}\right)$ to output the ciphertext σ as the reply to $A_{1}$’s query. When the $PID_{c}$’s public key is replaced, B may not be able to access the full private key of $PID_{c}$. In this case, the $A_{1}$ needs to provide the relevant information of the $PID_{c}$.
  $Q_{un}\left(\sigma ,ID_{u},PK_{u},PID_{c},PK_{c}\right)$: $A_{1}$ sends tuple $\left(\sigma ,ID_{u},PK_{u},PID_{c},PK_{c}\right)$ to B, where σ is the ciphertext intended for unsigncryption, $ID_{u}$ is the identity of U, $PK_{u}$ is the public key of U whose identity is $ID_{u}$, $PID_{c}$ is the identity of C and $PK_{c}$ is the public key of C whose identity is $PID_{c}$. B first executes the PPKG algorithm, SVS algorithm and FSKS algorithm using identity $ID_{u}$ to obtain $SK_{u}$, and then executes the unsigncrypt algorithm using the tuple $\left(\sigma ,ID_{u},SK_{u},PID_{c},PK_{c}\right)$ to obtain the plaintext *m* or ⊥ as the reply to $A_{1}$’s query. When the $ID_{u}$’s public key is replaced, B may not be able to access the full private key of $ID_{u}$. In this case, the $A_{1}$ needs to provide the relevant information of the $ID_{u}$.
- **Challenge phase**: $A_{1}$ selects two distinct messages $m_{0},m_{1}$ of the same length and subsequently transmits the tuple $\left(m_{0},m_{1},ID_{u}^{*},PK_{u}^{*},PID_{c}^{*},PK_{c}^{*}\right)$ to B, where $ID_{u}^{*}$ is the identity of U, $PK_{u}^{*}$ is the public key of U whose identity is $ID_{u}^{*}$, $PID_{c}^{*}$ is the identity of C and $PK_{c}^{*}$ is the public key of C whose identity is $PID_{c}^{*}$. B randomly chooses a bit ξ∈{0,1} and executes the signcryption algorithm using the tuple $\left(m_{\xi },ID_{u}^{*},PK_{u}^{*},PID_{c}^{*},PK_{c}^{*}\right)$ to obtain the ciphertext $\sigma^{*}$ of $m_{\xi }$. Then, B sends $\sigma^{*}$ to $A_{1}$. In this process, $A_{1}$ must meet the following conditions:
  (1) $ID_{u}^{*}$ is an identity whose partial private key has not been queried by $A_{1}$.
  (2) $A_{1}$ cannot replace the value of $R_{u}^{*}$.
- **Guess phase**: After receiving $\sigma^{*}$, $A_{1}$ performs a series of queries, but there are the following constraints:
  (1) $A_{1}$ is not allowed to operate $Q_{ppk}\left(ID_{u}^{*}\right)$.
  (2) $A_{1}$ is not allowed to replace the value of $R_{u}^{*}$.
  (3) $A_{1}$ is not allowed to operate $Q_{un}\left(\sigma^{*},ID_{u}^{*},ID_{u}^{*},PK_{u}^{*},PID_{c}^{*},PK_{c}^{*}\right)$.
  $A_{1}$ guesses $\xi^{\prime }$. If $\xi^{\prime }=\xi$, $A_{1}$ wins Game 1. The advantage of $A_{1}$ is defined as follows:
  $$Adv_{A_{1}}^{IND-CCA_{2}}=|P_{r}\left[\xi^{\prime }=\xi \right]-\frac{1}{2}|.$$

**Game 2**: The game between the adversary $A_{2}$ and the challenger B unfolds as follows:

- **Initialization phase**: $A_{2}$ obtains msk and params by executing the setup algorithm, then sends them to B.
- **Query phase**: $A_{2}$ performs various queries similar to Game 1.
- **Challenge phase**: $A_{2}$ selects two distinct messages $m_{0},m_{1}$ of the same length and subsequently transmits the tuple $\left(m_{0},m_{1},ID_{u}^{*},PK_{u}^{*},PID_{c}^{*},PK_{c}^{*}\right)$ to B, where $ID_{u}^{*}$ is the identity of U, $PK_{u}^{*}$ is the public key of U whose identity is $ID_{u}^{*}$, $PID_{c}^{*}$ is the identity of C and $PK_{c}^{*}$ is the public key of C whose identity is $PID_{c}^{*}$. B randomly chooses a bit ξ∈{0,1} and executes the signcryption algorithm using the tuple $\left(m_{\xi },ID_{u}^{*},PK_{u}^{*},PID_{c}^{*},PK_{c}^{*}\right)$ to obtain the ciphertext $\sigma^{*}$ of $m_{\xi }$. Then, B sends $\sigma^{*}$ to $A_{2}$. In this process, $A_{2}$ must meet the following conditions:
  (1) $ID_{u}^{*}$ is an identity whose secret value has not been queried by $A_{2}$.
  (2) $A_{2}$ is not allowed to replace the value of $X_{u}^{*}$.
- **Guess phase**: After receiving $\sigma^{*}$, $A_{2}$ performs a series of queries, but there are the following constraints:
  (1) $A_{2}$ is not allowed to operate $Q_{sv}\left(ID_{u}^{*}\right)$.
  (2) $A_{2}$ is not allowed to replace the value of $X_{u}^{*}$.
  (3) $A_{2}$ is not allowed to to operate $Q_{un}\left(\sigma^{*},ID_{u}^{*},ID_{u}^{*},PK_{u}^{*},PID_{c}^{*},PK_{c}^{*}\right)$.
  $A_{2}$ guesses $\xi^{\prime }$. If $\xi^{\prime }=\xi$, $A_{2}$ wins Game 2. The advantage of $A_{2}$ is defined as follows:
  $$Adv_{A_{2}}^{IND-CCA_{2}}=|P_{r}\left[\xi^{\prime }=\xi \right]-\frac{1}{2}|.$$

**Definition 6.**  *If the adversary cannot win the following game with a non-negligible probability in any polynomial time, then the security property of the CLSC scheme is said to satisfy unforgeability under an adaptive chosen message attack (UF-CMA).*

**Game 3**: The game between the adversary $A_{1}$ and the challenger B unfolds as follows:

- **Initialization phase:** Same as the initialization phase in Game 1.
- **Query phase:** $A_{1}$ performs various queries similar to Game 1.
- **Forgery phase:** $A_{1}$ outputs a new tuple $\left(\sigma^{*},ID_{u}^{*},PK_{u}^{*},PID_{c}^{*},PK_{c}^{*}\right)$, where $\sigma^{*}$ is a ciphertext, $ID_{u}^{*}$ is the identity of U, $PK_{u}^{*}$ is the public key of U whose identity is $ID_{u}^{*}$, $PID_{c}^{*}$ is the identity of C and $PK_{c}^{*}$ is the public key of C whose identity is $PID_{c}^{*}$. $A_{1}$ wins Game 3 if the subsequent conditions are met:
  (1) In the process of running the unsigncryption algorithm with the tuple $\left(\sigma^{*},ID_{u}^{*},SK_{u}^{*},PID_{c}^{*},PK_{c}^{*}\right)$, B does not output ⊥.
  (2) $A_{1}$ was not allowed to operate $Q_{ppk}\left(PID_{c}^{*}\right)$.
  (3) $A_{1}$ was not allowed to replace the value of $R_{c}^{*}$.
  (4) $A_{1}$ was not allowed to acquire $\sigma^{*}$ through running $Q_{sc}\left(m^{*},ID_{u}^{*},PK_{u}^{*},PID_{c}^{*},PK_{c}^{*}\right)$, where $m^{*}$ represents the plaintext that corresponds to $\sigma^{*}$.
  The advantage of $A_{1}$ is defined as follows:
  $$Adv_{A_{1}}^{UF-CMA}=|P_{r}\left[A_{1}wins\right]|.$$

**Game 4**: The game between the adversary $A_{2}$ and the challenger B unfolds as follows:

- **Initialization phase:** Same as the initialization phase in Game 2.
- **Query phase:** $A_{2}$ performs various queries similar to Game 1.
- **Forgery phase:** $A_{2}$ outputs a new tuple $\left(\sigma^{*},ID_{u}^{*},PK_{u}^{*},PID_{c}^{*},PK_{c}^{*}\right)$, where $\sigma^{*}$ is a ciphertext, $ID_{u}^{*}$ is the identity of U, $PK_{u}^{*}$ is the public key of U whose identity is $ID_{u}^{*}$, $PID_{c}^{*}$ is the identity of C and $PK_{c}^{*}$ is the public key of C whose identity is $PID_{c}^{*}$. $A_{2}$ wins Game 4 if the subsequent conditions are met:
  (1) In the process of running the unsigncryption algorithm with the tuple $\left(\sigma^{*},ID_{u}^{*},SK_{u}^{*},PID_{c}^{*},PK_{c}^{*}\right)$, B does not output ⊥.
  (2) $A_{2}$ was not allowed to operate $Q_{sv}\left(PID_{c}^{*}\right)$.
  (3) $A_{2}$ was not allowed to replace the value of $X_{c}^{*}$.
  (4) $A_{2}$ was not allowed to acquire $\sigma^{*}$ through running $Q_{sc}\left(m^{*},ID_{u}^{*},PK_{u}^{*},PID_{c}^{*},PK_{c}^{*}\right)$, where $m^{*}$ represents the plaintext that corresponds to $\sigma^{*}$.
  The advantage of $A_{2}$ is defined as follows:
  $$Adv_{A_{2}}^{UF-CMA}=|P_{r}\left[A_{2}wins\right]|.$$

**Definition 7.**  *If the adversary cannot win the following game with a non-negligible probability in any polynomial time, then the CLSC scheme is said to be anonymous to the signcrypter.*

**Game 5**: The game between the super adversary A and the challenger B unfolds as follows:

- **Initialization phase:** Same as the initialization phase in Game 2.
- **Query phase:** A inputs various queries, and B executes the corresponding algorithm to output the answer.
- **Challenge phase:** A selects a message $m^{*}$ and two distinct real identities $ID_{0}^{*}$ and $ID_{1}^{*}$ of C, where A has not performed $Q_{pid}$ for $ID_{0}^{*}$ and $ID_{1}^{*}$. A subsequently sends tuple $\left(m^{*},ID_{0}^{*},ID_{1}^{*},ID_{u}^{*},PK_{u}^{*}\right)$ to B, where $ID_{u}^{*}$ is the identity of U, $PK_{u}^{*}$ is the public key of U whose identity is $ID_{u}^{*}$. B performs the subsequent steps:
  (1) Randomly selects ξ∈{0,1} and invokes the PIDG algorithm with $ID_{\xi }^{*}$ to acquire $PID_{\xi }^{*}$.
  (2) Invokes the PPKG algorithm, SVS algorithm and FSKS algorithm with $PID_{\xi }^{*}$ to acquire the full private key $SK_{\xi }^{*}$ of $PID_{\xi }^{*}$.
  (3) Acquires the ciphertext $\sigma^{*}$ by running the signcryption algorithm with the tuple $\left(m^{*},ID_{u}^{*},PK_{u}^{*},PID_{\xi }^{*},SK_{\xi }^{*}\right)$.
  (4) Outputs the tuple $\left(\sigma^{*},ID_{u}^{*},PK_{u}^{*},PID_{\xi }^{*},PK_{\xi }^{*}\right)$ to A.
- **Guess phase:** A can make a series of queries, but cannot perform $Q_{pid}$ for $ID_{1}^{*}$ and $ID_{0}^{*}$. A makes a guess $\xi^{\prime }$. If $\xi^{\prime }=\xi$, A will win Game 5.
  The advantage of A is as follows:
  $$Adv_{A}^{ANO-CLSC}=|P_{r}\left[\xi^{\prime }=\xi \right]-1|.$$

**Definition 8.**  *If KGC can recognize the true identity of C in any ciphertext, then the CLSC scheme is identifiable.*

## 5. New Scheme

- Setup: Given a security parameter μ, SP performs the subsequent steps:
  (1) Sets up a bilinear mapping $e:G_{1}\times G_{1}\to G_{2}$, where $G_{1}$ is an additive cyclic group, $G_{2}$ is a multiplicative cyclic group and $|G_{1}\left|=\right|G_{2}|=q$($q>2^{\mu }$).
  (2) Selects a generator *P* of $G_{1}$, and computes N=e(P,P).
  (3) Sets an identity space $\Omega ={\left\{0,1\right\}}^{l_{1}}$ and a message space $M={\left\{0,1\right\}}^{l_{2}}$.
  (4) Selects the following secure hash functions (where $G_{1}^{2}=G_{1}\times G_{1}$).
  $H_{1}$: $G_{1}\times G_{1}\times G_{1}\to {\left\{0,1\right\}}^{l_{1}}$;
  $H_{2}$: ${\left\{0,1\right\}}^{l_{1}}\times G_{1}\to Z_{q}^{*}$;
  $H_{3}$: $G_{1}\times {\left\{0,1\right\}}^{l_{1}}\times G_{1}^{2}\times {\left\{0,1\right\}}^{l_{1}}\times G_{1}^{2}\to Z_{q}^{*}$;
  $H_{4}$: ${\left\{0,1\right\}}^{l_{2}}\times G_{1}\times {\left\{0,1\right\}}^{l_{1}}\times G_{1}^{2}\times {\left\{0,1\right\}}^{l_{1}}\times G_{1}^{2}\to Z_{q}^{*}$;
  $H_{5}$: $G_{1}\times G_{1}\times {\left\{0,1\right\}}^{l_{1}}\times G_{1}^{2}\times {\left\{0,1\right\}}^{l_{1}}\times G_{1}^{2}\to Z_{q}^{*}$.
  (5) Randomly chooses a number $\delta \in Z_{q}^{*}$ and computes $P_{pub}=\delta P$; let master secret key msk={δ}.
  (6) Publishes the params $params=\{G_{1},G_{2},q,e,P,P_{pub},N,H_{1}∼H_{5}\}$.
- PIDG: KGC sets up a list FI, which contains the tuple $\left(PID_{c},e_{c},E_{c},f_{c},F_{c}\right)$. Upon receiving an actual identity $ID_{c}\in \Omega$, KGC performs the subsequent steps:
  (1) Randomly chooses $e_{c},f_{c}\in_{R}Z_{q}^{*}$, and calculates $E_{c}=e_{c}P$, $F_{c}=f_{c}P$.
  (2) Computes $▵C=e_{c}f_{c}\delta P$, $PID_{c}=ID_{c}⊕H_{1}\left(▵C,E_{c},F_{c}\right)$.
  (3) Sends the pseudo-identity $PID_{c}$ to C.
  (4) Adds the tuple $\left(PID_{c},e_{c},E_{c},f_{c},F_{c}\right)$ to the list FI.
- PPKG:
  (1) After receiving the pseudo-identity $PID_{c}$ of C, KGC performs the subsequent steps:
    (a) Randomly chooses $r_{c}\in Z_{q}^{*}$, and calculates $R_{c}=r_{c}P$.
    (b) Calculates $l_{c}=H_{2}\left(PID_{c},R_{c}\right),d_{c}=r_{c}+l_{c}\delta$.
    (c) Sends $\left(R_{c},d_{c}\right)$ to C via a secure channel.
  (2) After receiving the identity $ID_{u}$ of U, KGC performs the subsequent steps:
    (a) Randomly chooses $r_{u}\in Z_{q}^{*}$, and calculates $R_{u}=r_{u}P$.
    (b) Calculates $l_{u}=H_{2}\left(ID_{u},R_{u}\right)$, $d_{u}=r_{u}+l_{u}\delta$.
    (c) Sends $\left(R_{u},d_{u}\right)$ to U via a secure channel.
  (3) C can confirm $d_{c}$’s validity by verifying whether the equation $d_{c}P=R_{c}+l_{c}P_{pub}$ holds. If the equation holds, then the partial private key is valid. Otherwise, the partial private key is invalid.
  (4) U can confirm $d_{u}$’s validity by verifying whether the equation $d_{u}P=R_{u}+l_{u}P_{pub}$ holds. If the equation holds, then the partial private key is valid. Otherwise, the partial private key is invalid.
- SVS:
  (1) C randomly chooses $x_{c}\in Z_{q}^{*}$, sets $x_{c}$ as its secret value.
  (2) U randomly chooses $x_{u}\in Z_{q}^{*}$, sets $x_{u}$ as its secret value.
- FSKS:
  (1) C sets the full private key $SK_{c}=\left(x_{c},d_{c}\right)$.
  (2) U sets the full private key $SK_{u}=\left(x_{u},d_{u}\right)$.
- UPKG:
  (1) C computes $X_{c}=x_{c}P$, and sets the public key $PK_{c}=\left(X_{c},R_{c}\right)$.
  (2) U computes $X_{u}=x_{u}P$, and sets the public key $PK_{u}=\left(X_{u},R_{u}\right)$.
- Signcrypt: Upon receiving a plaintext message m∈M, C performs the subsequent steps:
  (1) Calculates $l_{u}=H_{2}\left(ID_{u},R_{u}\right)$.
  (2) Randomly chooses $k\in Z_{q}^{*}$, and calculates K=kP.
  (3) Calculates $\lambda =H_{3}\left(K,ID_{u},PK_{u},PID_{c},PK_{c}\right)$.
  (4) Calculates $\tau =k(R_{u}+l_{u}P_{pub}+\lambda X_{u})$.
  (5) Calculates $\theta =H_{5}\left(K,\tau ,ID_{u},PK_{u},PID_{c},PK_{c}\right)⊕m$.
  (6) Calculates $\rho =H_{4}\left(\theta ,K,ID_{u},PK_{u},PID_{c},PK_{c}\right)$.
  (7) Calculates $\omega =\frac{1}{d_{c}+\rho x_{c}}P$.
  (8) Generates σ=(θ,K,ω) as the ciphertext.
  (9) Transmits σ to U.
- Unsigncrypt: Upon receiving the tuple σ=(θ,K,ω), U performs the subsequent steps:
  (1) Calculates $l_{c}=H_{2}\left(PID_{c},R_{c}\right)$.
  (2) Calculates $\rho =H_{4}\left(\theta ,K,ID_{u},PK_{u},PID_{c},PK_{c}\right)$.
  (3) Verifies whether the equation $e(\omega ,R_{c}+l_{c}P_{pub}+\rho X_{c})=N$ holds. If the equation is valid, proceed to step 4. Otherwise, the signature is invalid; output ⊥.
  (4) Calculates $\lambda =H_{3}\left(K,ID_{u},PK_{u},PID_{c},PK_{c}\right)$.
  (5) Calculates $\tau =(d_{u}+\lambda x_{u})K$.
  (6) Calculates $m=H_{5}\left(K,\tau ,ID_{u},PK_{u},PID_{c},PK_{c}\right)⊕\theta$.

**Correctness:**

$$\begin{array}{cc} \tau & =(d_{u}+\lambda x_{u})K\\ \; & =(d_{u}+\lambda x_{u})kP\\ \; & =k(d_{u}+\lambda x_{u})P\\ \; & =k(R_{u}+l_{u}P_{pub}+\lambda X_{u}) \end{array}$$

$$\begin{array}{cc} e(\omega ,R_{c}+l_{c}P_{pub}+\rho X_{c}) & =e(\frac{1}{d_{c}+\rho x_{c}}P,r_{c}P+l_{c}\delta P+\rho x_{c}P)\\ \; & =e(\frac{1}{r_{c}+l_{c}\delta +\rho x_{c}}P,\left(r_{c}+l_{c}\delta +\rho x_{c}\right)P)\\ \; & =e(\frac{1}{r_{c}+l_{c}\delta +\rho x_{c}}P,\left(r_{c}+l_{c}\delta +\rho x_{c}\right)P)\\ \; & =e(P,P)\\ \; & =N \end{array}$$

Additionally, our scheme offers public verifiability and public ciphertext authenticity. During the initial three steps of Unsigncrypt algorithm, any third party can ascertain the legitimacy of the ciphertext σ without needing C’s full private key or the message *m*. If the ciphertext σ is proven invalid, the receiver can immediately disregard it, thus avoiding further decryption steps. This method conserves computational resources and reduces energy consumption, which is particularly advantageous for small-scale devices by saving both energy and processing time.

From an ethical perspective, we have conducted an analysis of the ethical risks associated with the proposed scheme and its security model. This analytical framework primarily encompasses three core aspects: technical ethics, individual ethics, and social ethics. In terms of technical ethics, we have provided a more robust security model for the CL-ASC scheme. Our security model considers a malicious yet passive KGC as a Type II adversary and allows for such adversaries to replace public keys. Both Type I and Type II adversaries are capable of directly computing hash functions to obtain results. This significantly enhances the adversaries’ capabilities, thereby making the scheme more secure. Under our enhanced security model, we will demonstrate that the CL-ASC scheme possesses indistinguishability and unforgeability. Consequently, applying our CL-ASC scheme for communication in WBNA will not result in message leakage. Furthermore, our CL-ASC scheme ensures the anonymity of the signcrypter, effectively safeguarding users’ privacy. In individual ethics, the ciphertext of signcryption is encrypted with the sender’s private key and the recipient’s public key. To unsigncrypt, the recipient’s private key and the sender’s public key are required. This ensures that even if a participant is subjected to malicious attacks during data transmission, the transmitted data will not be leaked, thus avoiding the risk of individual ethics. In terms of social ethics: encryption measures are taken for users’ private data during the communication process. When strictly implemented, our scheme can maximize the prevention of data leakage during transmission.

## 6. Security of the Scheme

In the security proofs below, the adversary is capable of directly computing the values of the hash function without necessitating a query to the challenger.

**Lemma 1.**  *If the DDH problem is hard, our scheme is proven to be $IND-CCA_{2}$ against the adversary $A_{1}$ in the SM.*

**Proof.**  Given the tuple (P,αP,βP,T), where $\alpha ,\beta \in Z_{q}^{*}$ and α,β are unknown. The goal of B is to determine whether *T* is equal to αβP.

**Initialization phase:** B obtains msk and $params=\{G_{1},G_{2},q,e,P,P_{pub}=\delta P,N=e\left(P,P\right),H_{1}∼H_{5}\}$ by executing the setup algorithm, then sends params to $A_{1}$ and maintains the secrecy of msk. After the process above, $A_{1}$ and B are both unaware of α and β, but B is aware of δ, while $A_{1}$ is not.

**Query phase:** B sets $ID^{♢}$ as the challenge target identity. For C, $A_{1}$ must first execute $Q_{pid}\left(ID_{c}\right)$ and $Q_{upk}\left(PID_{c}\right)$ before any other queries. For U, $A_{1}$ must first execute $Q_{upk}\left(ID_{u}\right)$ before any other queries. There are eight empty tables, $L_{UC},L_{UU},L_{RC},L_{RU},L_{KC},L_{KU},L_{VC}$ and $L_{VU}$, maintained by B. $A_{1}$ can conduct the following types of queries, and B simulates $A_{1}$’s queries as follows:

$Q_{pid}\left(ID_{c}\right)$: When $A_{1}$ provides an identity $ID_{c}$ for a query, B executes the PIDG algorithm to output the $PID_{c}$ as $A_{1}$’s response.

$Q_{upk}\left(PID_{c}\right)$: B maintains a list $L_{UC}$, which contains the tuple $\left(PID_{c},X_{c},x_{c},R_{c},r_{c}\right)$. When $A_{1}$ provides an identity $PID_{c}$ for a query, if the $PID_{c}$ is on the the list $L_{UC}$, B returns $PK_{c}$ as $A_{1}$’s response. Otherwise, $PID_{c}$ is queried as a new identity, B randomly chooses $x_{c},r_{c}\in Z_{q}^{*}$, sets $PK_{c}=\left(x_{c}P,r_{c}P\right)$ as $A_{1}$’s response, and adds $\left(PID_{c},x_{c}P,x_{c},r_{c}P,r_{c}\right)$ to the list $L_{UC}$.

$Q_{upk}\left(ID_{u}\right)$: B maintains a list $L_{UU}$, which contains the tuple $\left(ID_{u},X_{u},x_{u},R_{u},r_{u}\right)$. When $A_{1}$ provides an identity $ID_{u}$ for a query, if the $ID_{u}$ is on the the list $L_{UU}$, B returns $PK_{u}$ as $A_{1}$’s response. Otherwise, $ID_{u}$ is queried as a new identity, and B performs the subsequent steps:

(1) If $ID_{u}=ID^{♢}$, B randomly chooses $x^{♢}\in Z_{q}^{*}$, sets $PK_{u}=PK^{♢}=\left(x^{♢}P,\alpha P\right)$ as $A_{1}$’s response, and adds $\left(ID_{u},x^{♢}P,x^{♢},\alpha P,\nabla \right)$ to the list $L_{UU}$(where ∇ represents a null value).

(2) If $ID_{u}\neq ID^{♢}$, B randomly chooses $x_{u},r_{u}\in Z_{q}^{*}$, computes $PK_{u}=\left(x_{u}P,r_{u}P\right)$ as $A_{1}$’s response, and adds $\left(ID_{u},x_{u}P,x_{u},r_{u}P,r_{u}\right)$ to the list $L_{UU}$.

$R_{upk}\left(PID_{c},PK_{c},PK_{c}^{\prime }\right)$: B maintains a list $L_{RC}$, which contains the tuple $\left(PID_{c},PK_{c},PK_{c}^{\prime }\right)$. When $A_{1}$ requests to replace the $PID_{C}$’s public key $PK_{C}$ with $PK_{c}^{\prime }$, B updates $PK_{c}$ to $PK_{c}^{\prime }$, and adds $\left(PID_{c},PK_{c},PK_{c}^{\prime }\right)$ to the list $L_{RC}$.

$R_{upk}\left(ID_{u},PK_{u},PK_{u}^{\prime }\right)$: B maintains a list $L_{RU}$, which contains the tuple $\left(ID_{u},PK_{u},PK_{u}^{\prime }\right)$. When $A_{1}$ requests to replace the $ID_{u}$’s public key $PK_{u}$ with $PK_{u}^{\prime }$, B updates $PK_{u}$ to $PK_{u}^{\prime }$, and adds $\left(ID_{u},PK_{u},PK_{u}^{\prime }\right)$ to the list $L_{RU}$.

$Q_{ppk}\left(PID_{c}\right)$: B maintains a list $L_{KC}$, which contains the tuple $\left(PID_{c},d_{c}\right)$. When $A_{1}$ provides an identity $PID_{c}$ for a query, B searches for $\left(PID_{c},x_{c}P,x_{c},r_{c}P,r_{c}\right)$ in the list $L_{UC}$, executes the PPKG algorithm, and outputs $d_{c}$ as $A_{1}$’s response, then adds $\left(PID_{c},d_{c}\right)$ to the list $L_{KC}$.

$Q_{ppk}\left(ID_{u}\right)$: B maintains a list $L_{KU}$, which contains the tuple $\left(ID_{u},d_{u}\right)$. When $A_{1}$ provides an identity $PID_{c}$ for a query, B performs the subsequent steps:

(1) If $ID_{u}=ID^{♢}$, then B fails and terminates the process.

(2) If $ID_{u}\neq ID^{♢}$, B searches for $\left(ID_{u},x_{u}P,x_{u},r_{u}P,r_{u}\right)$ in the list $L_{UU}$, executes the PPKG algorithm to output $d_{u}$ as $A_{1}$’s response, and then adds $\left(ID_{u},d_{u}\right)$ to the list $L_{KU}$.

$Q_{sv}\left(PID_{c}\right)$: B maintains a list $L_{VC}$, which contains the tuple $\left(PID_{c},x_{c}\right)$. When $A_{1}$ provides an identity $PID_{c}$ for a query, B searches for $\left(PID_{c},x_{c}P,x_{c},r_{c}P,r_{c}\right)$ in the list $L_{UC}$, outputs $x_{c}$ as $A_{1}$’s response, and then adds $\left(PID_{c},x_{c}\right)$ to the list $L_{VC}$.

$Q_{sv}\left(ID_{u}\right)$: B maintains a list $L_{VU}$, which contains the tuple $\left(ID_{u},x_{u}\right)$. When $A_{1}$ provides an identity $ID_{u}$ for a query, B searches for $\left(ID_{u},x_{u}P,x_{u},r_{u}P,r_{u}\right)$ in the list $L_{UU}$, outputs $x_{u}$ as $A_{1}$’s response, and then adds $\left(ID_{u},x_{u}\right)$ to the list $L_{VU}$.

$Q_{sc}\left(m,ID_{u},PK_{u},PID_{c},PK_{c}\right)$: When $A_{1}$ provides tuple $\left(m,ID_{u},PK_{u},PID_{c},PK_{c}\right)$ for a query, B performs as follows:

(1) If $PID_{c}\in L_{RC}$, then $PK_{c}=\left(x_{c}P,r_{c}P\right)$ is replaced by $PK_{c}^{\prime }=\left(x_{c}^{\prime }P,r_{c}^{\prime }P\right)$. If $x_{u}^{\prime }\neq x_{u}$ (or $r_{u}^{\prime }\neq r_{u}$ ), $A_{1}$ must send $x_{c}^{\prime }$ (or $r_{c}^{\prime }$) to B. B first executes the PPKG algorithm and FSKS algorithm using identity $PID_{c}$ to obtain $SK_{c}$, and then executes the signcrypt algorithm with tuple $\left(m,ID_{u},PK_{u},PID_{c},SK_{c}\right)$ to output the ciphertext σ as $A_{1}$’s response.

(2) If $PID_{c}\notin L_{RC}$, B first executes the PPKG algorithm and FSKS algorithm using identity $PID_{c}$ to obtain $SK_{c}$, and then executes the signcrypt algorithm with tuple $\left(m,ID_{u},PK_{u},PID_{c},SK_{c}\right)$ to output the ciphertext σ as $A_{1}$’s response.

$Q_{un}\left(\sigma ,ID_{u},PK_{u},PID_{c},PK_{c}\right)$: When $A_{1}$ provides tuple $\left(\sigma ,ID_{u},PK_{u},PID_{c},PK_{c}\right)$ for a query, B performs as follows:

(1) If $ID_{u}\in L_{RU}$, then $PK_{u}=\left(x_{u}P,r_{u}P\right)$ is replaced by $PK_{u}^{\prime }=\left(x_{u}^{\prime }P,r_{u}^{\prime }P\right)$. If $x_{u}^{\prime }\neq x_{u}$ (or $r_{u}^{\prime }\neq r_{u}$ ), $A_{1}$ must send $x_{u}^{\prime }$ (or $r_{u}^{\prime }$) to B. B first executes the PPKG algorithm and FSKS algorithm using identity $ID_{u}$ to obtain $SK_{u}$, and then executes the unsigncryption algorithm with tuple $\left(\sigma ,ID_{u},SK_{u},PID_{c},PK_{c}\right)$ to output the plaintext *m* or ⊥ as $A_{1}$’s response.

(2) If $ID_{u}\notin L_{RU}$ and $ID_{u}\neq ID^{♢}$, B first executes the PPKG algorithm and FSKS algorithm using identity $ID_{u}$ to obtain $SK_{u}$, and then executes the unsigncryption algorithm with tuple $\left(\sigma ,ID_{u},SK_{u},PID_{c},PK_{c}\right)$ to output the plaintext *m* or ⊥ as $A_{1}$’s response.

(3) If $ID_{u}\notin L_{RU}$ and $ID_{u}=ID^{♢}$, B fails and terminates the process.

**Challenge phase:** $A_{1}$ selects two distinct messages $m_{0},m_{1}$ of the same length and subsequently transmits the tuple $\left(m_{0},m_{1},ID_{u}^{*},PK_{u}^{*},PID_{c}^{*},PK_{c}^{*}\right)$ to B. B performs the subsequent steps:

In Situation I, if $ID_{u}^{*}\neq ID^{♢}$, then B randomly chooses ξ∈{0,1} and performs $Q_{sc}\left(m_{\xi },ID_{u}^{*},PK_{u}^{*},PID_{c}^{*},PK_{c}^{*}\right)$ and outputs the ciphertext $\sigma^{*}$ to $A_{1}$.

In Situation II, if $ID_{u}^{*}=ID^{♢}$, B randomly chooses ξ∈{0,1} and performs the subsequent steps:

(1) Searches for $\left(PID_{c}^{*},x_{c}^{*}P,x_{c}^{*},r_{c}^{*}P,r_{c}^{*}\right)$ in the list $L_{UC}$.

(2) Sets $PK_{c}^{*}=\left(x_{c}^{*}P,r_{c}^{*}P\right)$.

(3) Calculates $l_{c}^{*}=H_{2}\left(PID_{c}^{*},R_{c}^{*}\right)$, $d_{c}^{*}=r_{c}^{*}+l_{c}^{*}\delta$.

(4) Searches for $\left(ID^{♢},x^{♢}P,x^{♢},\alpha P,\nabla \right)$ in the list $L_{UU}$.

(5) Sets $PK_{u}^{*}=PK^{♢}=\left(x^{♢}P,\alpha P\right)$.

(6) Calculates $l_{u}^{*}=H_{2}\left(ID^{♢},\alpha P\right)$.

(7) Sets $K^{*}=\beta P\left(k^{*}=\beta \right)$.

(8) Calculates $\lambda^{*}=H_{3}\left(K^{*},ID_{u}^{*},PK_{u}^{*},PID_{c}^{*},PK_{c}^{*}\right)$, $\tau^{*}=T+K^{*}\left(l_{u}^{*}\delta +\lambda^{*}x_{u}^{*}\right)$, $\theta^{*}=H_{5}\left(K^{*},\tau^{*},ID_{u}^{*},PK_{u}^{*},PID_{c}^{*},PK_{c}^{*}\right)⊕m_{\xi }$, $\rho^{*}=H_{4}\left(\theta^{*},K^{*},ID_{u}^{*},PK_{u}^{*},PID_{c}^{*},PK_{c}^{*}\right)$, and $\omega^{*}=\frac{1}{d_{c}^{*}+\rho^{*}x_{c}^{*}}P$.

(9) Outputs $\sigma^{*}=\left(\theta^{*},K^{*},\omega^{*}\right)$ to $A_{1}$.

**Guess phase:** $A_{1}$ performs various queries adaptively as in the query phase and follows the rules of Game 1. After that, $A_{1}$ outputs its guess $\xi^{\prime }\in \left\{0,1\right\}$.

**Solving the DDH problem**: B returns “1”, if $\xi^{\prime }=\xi$. Otherwise, B outputs “0”. If T=αβP, then

$$\begin{array}{cc} \tau^{*} & =\alpha \beta P+K^{*}\left(l_{u}^{*}\delta +\lambda^{*}x_{u}^{*}\right)\\ \; & =\beta (R_{u}^{*}+l_{u}^{*}P_{pub}+\lambda^{*}X_{u}^{*}) \end{array}$$

This means that $\sigma^{*}$ is a true ciphertext. Therefore, the advantage of $A_{1}$ in distinguishing symbol ξ is ε, that is to say:

$$\mathrm{Pr}\left[B\to 1|T=\alpha \beta P\right]=\mathrm{Pr}\left[\xi^{\prime }=\xi |T=\alpha \beta P\right]=\frac{1}{2}+\varepsilon .$$

If T≠αβP, then $\sigma^{*}$ is not a true ciphertext. This implies that for this $\sigma^{*}$, the distribution of ξ=0 and ξ=1 is the same. Therefore, $A_{1}$ cannot have any advantage in identifying symbol ξ, that is to say:

$$\mathrm{Pr}\left[B\to 1|T\neq \alpha \beta P\right]=\mathrm{Pr}\left[\xi^{\prime }=\xi |T\neq \alpha \beta P\right]=\frac{1}{2}.$$

Probability: Let $q_{UU}$, $q_{RU}$, $q_{KU}$ and $q_{UN}$ represent the number of $A_{1}$ executes $Q_{upk}\left(ID_{u}\right)$, $R_{upk}\left(ID_{u}\right)$, $Q_{ppk}\left(ID_{u}\right)$ and $Q_{un}\left(\sigma ,ID_{u},PK_{u},PID_{c},PK_{c}\right)$, respectively. Next, we will calculate the probability of B successfully solving a given DDH problem. To facilitate understanding, we defined the following three events:

$\pi_{1}$: $A_{1}$ has neither operated $Q_{ppk}\left(ID^{♢}\right)$ nor replaced the value of $R_{u}^{♢}\left(\alpha P\right)$.

$\pi_{2}$: $A_{1}$ has not failed in the $Q_{un}\left(\right)$.

$\pi_{3}$: $ID_{u}^{*}=ID^{♢}$.

Because if $A_{1}$ replaces the public key of $ID_{u}$, it cannot perform $Q_{ppk}\left(\right)$ for $ID_{u}$, therefore $L_{RU}\cap L_{KU}=\emptyset$. Based on the analysis, we can obtain the following results:

$$\begin{array}{cc} \; & \mathrm{Pr}\left[\pi_{1}\right]=\frac{q_{UU}-q_{RU}-q_{KU}}{q_{UU}}\\ \; & \mathrm{Pr}\left[\pi_{2}|\pi_{1}\right]={\left(1-\frac{1}{q_{UU}}\right)}^{q_{UN}}\approx e^{-\frac{q_{UN}}{q_{UU}}}\\ \; & \mathrm{Pr}\left[\pi_{3}|\pi_{1}\wedge \pi_{2}\right]=\frac{1}{q_{UN}-q_{RU}-q_{KU}} \end{array}$$

Then, the following results can be derived:

$$\begin{array}{cc} \mathrm{Pr}[B_{success}] & =\mathrm{Pr}[\pi_{1}\wedge \pi_{2}\wedge \pi_{3}]\\ \; & =\mathrm{Pr}\left[\pi_{1}\right]\mathrm{Pr}\left[\pi_{2}|\pi_{1}\right]\mathrm{Pr}\left[\pi_{3}|\pi_{1}\wedge \pi_{2}\right]\\ \; & \approx \frac{q_{UU}-q_{RU}-q_{KU}}{q_{UU}}e^{-\frac{q_{UN}}{q_{UU}}}\cdot \frac{1}{q_{UN}-q_{RU}-q_{KU}}\\ \; & \approx \frac{1}{q_{UU}}e^{-\frac{q_{UN}}{q_{UU}}} \end{array}$$

Consequently, if $A_{1}$ can distinguish symbol ξ with the advantage ε, then B can resolve the DDH problem with a probability of $\frac{\varepsilon }{q_{UU}}e^{-\frac{q_{UN}}{q_{UU}}}$. □

**Lemma 2.**  *If the DDH problem is hard, our scheme is proven to be $IND-CCA_{2}$ against the adversary $A_{2}$ in the SM.*

**Proof.**  Given the tuple (P,αP,βP,T), where $\alpha ,\beta \in Z_{q}^{*}$ and α,β are unknown. The goal of B is to determine whether *T* is equal to aβP.

**Initialization phase:** $A_{2}$ obtains msk and $params=\{G_{1},G_{2},q,e,P,P_{pub}=\delta P,N=e\left(P,P\right),H_{1}∼H_{5}\}$ by executing the setup algorithm, then sends them to B. After the process above, neither $A_{2}$ nor B knows α and β, but $A_{2}$ and B know δ.

**Query phase:** B sets $ID^{♢}$ as the challenge target identity. For C, $A_{2}$ must first execute $Q_{pid}\left(ID_{c}\right)$ and $Q_{upk}\left(PID_{c}\right)$ before any other queries. For U, $A_{2}$ must first execute $Q_{upk}\left(ID_{u}\right)$ before any other queries. There are eight empty tables, $L_{UC},L_{UU},L_{RC},L_{RU},L_{KC},L_{KU},L_{VC}$ and $L_{VU}$, maintained by B. $A_{2}$ can conduct the following types of queries, and B simulates $A_{2}$’s queries as follows:

$Q_{pid}\left(ID_{c}\right)$: Similar to Lemma 1.

$Q_{upk}\left(PID_{c}\right)$: Similar to Lemma 1.

$Q_{upk}\left(ID_{u}\right)$: B maintains a list $L_{UU}$, which includes the the tuple $\left(ID_{u},X_{u},x_{u},R_{u},r_{u}\right)$. When $A_{2}$ provides an identity $ID_{u}$ for a query, if the $ID_{u}$ is on the the list $L_{UU}$, B returns $PK_{u}$ as $A_{2}$’s response. Otherwise, $ID_{u}$ is queried as a new identity, B performs the subsequent steps:

(1) If $ID_{u}=ID^{♢}$, B randomly chooses $r^{♢}\in Z_{q}^{*}$, sets $PK_{u}=PK^{♢}=\left(\alpha P,r^{♢}P\right)$ as $A_{2}$’s response, and adds $\left(ID^{♢},\alpha P,\nabla ,r^{♢}P,r^{♢}\right)$ to the list $L_{UU}$(where ∇ represents a null value).

(2) If $ID_{u}\neq ID^{♢}$, B randomly chooses $x_{u},r_{u}\in Z_{q}^{*}$, computes $PK_{u}=\left(x_{u}P,r_{u}P\right)$ as $A_{2}$’s response, and adds $\left(ID_{u},x_{u}P,x_{u},r_{u}P,r_{u}\right)$ to the list $L_{UU}$.

$R_{upk}\left(PID_{c},PK_{c}^{\prime }\right)$: Similar to Lemma 1.

$R_{upk}\left(ID_{u},PK_{u}^{\prime }\right)$: Similar to Lemma 1.

$Q_{ppk}\left(PID_{c}\right)$: Similar to Lemma 1.

$Q_{ppk}\left(ID_{u}\right)$: B maintains a list $L_{KU}$, which contains the tuple $\left(ID_{u},d_{u}\right)$. When $A_{2}$ provides an identity $ID_{u}$ for a query, B searches for $\left(ID_{u},x_{u}P,x_{u},r_{u}P,r_{u}\right)$ in the list $L_{UU}$, and then executes PPKG algorithm to output the tuple $d_{u}$. After that, B adds $\left(ID_{u},d_{u}\right)$ to the list $L_{KU}$.

$Q_{sv}\left(PID_{c}\right)$: Similar to Lemma 1.

$Q_{sv}\left(ID_{u}\right)$: B maintains the list $L_{VU}$, which contains the tuple $\left(ID_{u},x_{u}\right)$. When $A_{2}$ provides an identity $ID_{u}$ for a query, B performs the subsequent steps:

(1) If $ID_{u}=ID^{♢}$, then B fails and terminates the process.

(2) If $ID_{u}\neq ID^{♢}$, B searches for $\left(ID_{u},x_{u}P,x_{u},r_{u}P,r_{u}\right)$ in the list $L_{UU}$, outputs $x_{u}$ as $A_{2}$’s response, and then adds $\left(ID_{u},x_{u}\right)$ to the list $L_{VU}$.

$Q_{sc}\left(m,ID_{u},PK_{u},PID_{c},PK_{c}\right)$: Similar to Lemma 1.

$Q_{un}\left(\sigma ,ID_{u},PK_{u},PID_{c},PK_{c}\right)$: Similar to Lemma 1.

**Challenge phase**: $A_{2}$ selects two distinct messages $m_{0},m_{1}$ of the same length and subsequently transmits the tuple $\left(m_{0},m_{1},ID_{u}^{*},PK_{u}^{*},PID_{c}^{*},PK_{c}^{*}\right)$ to B. B performs the subsequent steps:

In Situation I, if $ID_{u}^{*}\neq ID^{♢}$, B randomly chooses ξ∈{0,1} and performs $Q_{sc}\left(m_{\xi },ID_{u}^{*},PK_{u}^{*},PID_{c}^{*},PK_{c}^{*}\right)$ to output the ciphertext $\sigma^{*}$ to $A_{2}$.

In Situation II, if $ID_{u}^{*}=ID^{♢}$, B randomly chooses ξ∈{0,1} and performs the subsequent steps:

(1) Searches for $\left(PID_{c}^{*},x_{c}^{*}P,x_{c}^{*},r_{c}^{*}P,r_{c}^{*}\right)$ in the list $L_{UC}$.

(2) Sets $PK_{c}^{*}=\left(x_{c}^{*}P,r_{c}^{*}P\right)$.

(3) Calculates $l_{c}^{*}=H_{2}\left(PID_{c}^{*},R_{c}^{*}\right)$, $d_{c}^{*}=r_{c}^{*}+l_{c}^{*}\delta$.

(4) Searches for $\left(ID^{♢},\alpha P,\nabla ,r^{♢}P,r^{♢}\right)$ in the list $L_{UU}$.

(5) Sets $PK_{u}^{*}=PK^{♢}=\left(\alpha P,r^{♢}P\right)$.

(6) Calculates $l_{u}^{*}=H_{2}\left(ID^{♢},r^{♢}P\right)$, $d_{u}^{*}=r_{u}^{*}+l_{u}^{*}\delta$ (where $r_{u}^{*}=r^{♢}$ ).

(7) Sets $K^{*}=\beta P\left(k^{*}=\beta \right)$.

(8) Calculates $\lambda^{*}=H_{3}\left(K^{*},ID_{u}^{*},PK_{u}^{*},PID_{c}^{*},PK_{c}^{*}\right)$, $\tau^{*}=\lambda^{*}T+\left(r_{u}^{*}+l_{u}^{*}\delta \right)K^{*}$, $\theta^{*}=H_{5}\left(K^{*},\tau^{*},ID_{u}^{*},PK_{u}^{*},PID_{c}^{*},PK_{c}^{*}\right)⊕m_{\xi }$, $\rho^{*}=H_{4}\left(\theta^{*},K^{*},ID_{u}^{*},PK_{u}^{*},PID_{c}^{*},PK_{c}^{*}\right)$, and $\omega^{*}=\frac{1}{d_{c}^{*}+\rho^{*}x_{c}^{*}}P$.

(9) Outputs $\sigma^{*}=\left(\theta^{*},K^{*},\omega^{*}\right)$ to $A_{2}$.

**Guess phase**: $A_{2}$ performs various queries adaptively, as in the query phase, and follows the rules of Game 2. After that, $A_{2}$ outputs its guess $\xi^{\prime }\in \left\{0,1\right\}$.

**Solving the DDH problem**: B returns “1”, if $\xi^{\prime }=\xi$. Otherwise, B outputs “0”. If T=αβP, then

$$\begin{array}{cc} \tau^{*} & =\lambda^{*}\alpha \beta P+\left(r_{u}^{*}+l_{u}^{*}\delta \right)K^{*}\\ \; & =\beta (r_{u}^{*}+l_{u}^{*}\delta +\lambda^{*}\alpha )P\\ \; & =\beta (R_{u}^{*}+l_{u}^{*}P_{pub}+\lambda^{*}X_{u}^{*}) \end{array}$$

This means that $\sigma^{*}$ is a true ciphertext. Therefore, the advantage of $A_{2}$ in distinguishing symbol ξ is ε, that is to say:

$$\mathrm{Pr}\left[B\to 1|T=\alpha \beta P\right]=\mathrm{Pr}\left[\xi^{\prime }=\xi |T=\alpha \beta P\right]=\frac{1}{2}+\varepsilon .$$

If T≠αβP, then $\sigma^{*}$ is not a true ciphertext. This implies that for this $\sigma^{*}$, the distribution of ξ=0 and ξ=1 is the same. Therefore, $A_{2}$ cannot have any advantage in identifying symbol ξ, that is to say:

$$\mathrm{Pr}\left[B\to 1|T\neq \alpha \beta P\right]=\mathrm{Pr}\left[\xi^{\prime }=\xi |T\neq \alpha \beta P\right]=\frac{1}{2}.$$

Probability: Let $q_{UU}$, $q_{RU}$, $q_{VU}$ and $q_{UN}$ represent the number of $A_{2}$ executes $Q_{upk}\left(ID_{u}\right)$, $R_{upk}\left(ID_{u}\right)$, $Q_{sv}\left(ID_{u}\right)$ and $Q_{un}\left(\sigma ,ID_{u},PK_{u},PID_{c},PK_{c}\right)$, respectively. Next, we will calculate the probability of B successfully solving a given DDH problem. To facilitate understanding, we defined the following three events:

$\pi_{1}$: $A_{2}$ has neither operated $Q_{sv}\left(ID^{♢}\right)$ nor replaced the value of $X_{u}^{♢}\left(\alpha P\right)$.

$\pi_{2}$: $A_{2}$ has not failed in the $Q_{un}\left(\right)$.

$\pi_{3}$: $ID_{u}^{*}=ID^{♢}$.

Because if $A_{2}$ replaces the public key of $ID_{u}$, it cannot perform $Q_{sv}\left(\right)$ for $ID_{u}$, therefore $L_{RU}\cap L_{VU}=\emptyset$. Based on the analysis, we can obtain the following results:

$$\begin{array}{cc} \; & \mathrm{Pr}\left[\pi_{1}\right]=\frac{q_{UU}-q_{RU}-q_{VU}}{q_{UU}}\\ \; & \mathrm{Pr}\left[\pi_{2}|\pi_{1}\right]={\left(1-\frac{1}{q_{UU}}\right)}^{q_{UN}}\approx e^{\frac{q_{UN}}{q_{UU}}}\\ \; & \mathrm{Pr}\left[\pi_{3}|\pi_{1}\wedge \pi_{2}\right]=\frac{1}{q_{UN}-q_{RU}-q_{VU}} \end{array}$$

Then, the following results can be derived:

$$\begin{array}{cc} \mathrm{Pr}[B_{success}] & =\mathrm{Pr}[\pi_{1}\wedge \pi_{2}\wedge \pi_{3}]\\ \; & =\mathrm{Pr}\left[\pi_{1}\right]\mathrm{Pr}\left[\pi_{2}|\pi_{1}\right]\mathrm{Pr}\left[\pi_{3}|\pi_{1}\wedge \pi_{2}\right]\\ \; & \approx \frac{q_{UU}-q_{RU}-q_{VU}}{q_{UU}}e^{-\frac{q_{UN}}{q_{UU}}}\cdot \frac{1}{q_{UN}-q_{RU}-q_{VU}}\\ \; & \approx \frac{1}{q_{UU}}e^{-\frac{q_{UN}}{q_{UU}}} \end{array}$$

Consequently, if $A_{2}$ can distinguish symbol ξ with the advantage ε, then B can resolve the DDH problem with a probability of $\frac{\varepsilon }{q_{UU}}e^{-\frac{q_{UN}}{q_{UU}}}$. □

**Theorem 1.**  *If the DDH problem is hard, our scheme is proven to be $IND-CCA_{2}$ in the SM.*

**Proof.**  From Lemmas 1 and 2, we can see that the conclusion is correct. □

**Lemma 3.**  *If the CCA problem is hard, our scheme is proven to be UF-CMA against the adversary $A_{1}$ in the SM.*

**Proof.**  Given the tuple (P,αP). The goal of B is to output the tuple $\left(\gamma ,\frac{1}{\alpha +\gamma }P\right)$.

**Initialization phase:** Same as the initialization phase in Lemma 1.

**Query phase:** B sets $PID^{♢}$ as the challenge target identity. $A_{1}$ can conduct the following types of queries, and B simulates $A_{1}$’s queries as follows:

$Q_{pid}\left(ID_{c}\right)$: Similar to Lemma 1.

$Q_{upk}\left(PID_{c}\right)$: B maintains a list $L_{UC}$, which includes the tuple $\left(PID_{c},X_{c},x_{c},R_{c},r_{c}\right)$. When $A_{1}$ provides an identity $PID_{c}$ for a query, if the $PID_{c}$ is on the list $L_{UC}$, B returns $PK_{c}$ as $A_{1}$’s response. Otherwise, $PID_{c}$ is queried as a new identity, B performs the subsequent steps:

(1) If $PID_{c}=ID^{♢}$, B randomly chooses $x^{♢}\in Z_{q}^{*}$, sets $PK_{c}=PK^{♢}=\left(x^{♢}P,\alpha P\right)$ as $A_{1}$’s response, and adds the tuple $\left(PID^{♢},x^{♢}P,x^{♢},\alpha P,\nabla \right)$ to the list $L_{UC}$ (where ∇ represents a null value).

(2) If $PID_{c}\neq ID^{♢}$, B randomly chooses $x_{c},r_{c}\in Z_{q}^{*}$, sets $PK_{c}=\left(x_{c}P,r_{c}P\right)$ as $A_{1}$’s response, and adds the tuple $\left(PID_{c},x_{c}P,x_{c},r_{c}P,r_{c}\right)$ to the list $L_{UC}$.

$Q_{upk}\left(ID_{u}\right)$: B maintains a list $L_{UU}$, which contains the tuple $\left(ID_{u},X_{u},x_{u},R_{u},r_{u}\right)$. When $A_{1}$ provides an identity $ID_{u}$ for a query, if the $ID_{u}$ is on the the list $L_{UU}$, B returns $PK_{u}$ as $A_{1}$’s response. Otherwise, $ID_{u}$ is queried as a new identity, B randomly chooses $x_{u},r_{u}\in Z_{q}^{*}$, sets $PK_{u}=\left(x_{u}P,r_{u}P\right)$, and adds $\left(ID_{u},x_{u}P,x_{u},r_{u}P,r_{u}\right)$ to the list $L_{UU}$.

$R_{upk}\left(PID_{c},PK_{c}^{\prime }\right)$: Similar to Lemma 1.

$R_{upk}\left(ID_{u},PK_{u}^{\prime }\right)$: Similar to Lemma 1.

$Q_{ppk}\left(PID_{c}\right)$: B maintains a list $L_{KC}$, which includes the tuple $\left(PID_{c},d_{c}\right)$. When $A_{1}$ provides an identity $PID_{c}$ for a query, B performs the subsequent steps:

(1) If $PID_{c}=PID^{♢}$, then B fails and terminates the process.

(2) If $PID_{c}\neq PID^{♢}$, B searches for $\left(PID_{c},x_{c}P,x_{c},r_{c}P,r_{c}\right)$ in the list $L_{UC}$, executes the PPKG algorithm to output $d_{c}$ as $A_{1}$’s response, and then adds $\left(PID_{c},d_{c}\right)$ to the list $L_{UC}$.

$Q_{ppk}\left(ID_{u}\right)$: Similar to Lemma 2.

$Q_{sv}\left(PID_{c}\right)$: Similar to Lemma 1.

$Q_{sv}\left(ID_{u}\right)$: Similar to Lemma 1.

$Q_{sc}\left(m,ID_{u},PK_{u},PID_{c},PK_{c}\right)$: When $A_{1}$ provides tuple $\left(m,ID_{u},PK_{u},PID_{c},PK_{c}\right)$ for a query, B performs as follows:

(1) If $PID_{c}\in L_{RC}$, then $PK_{c}=\left(x_{c}P,r_{c}P\right)$ is replaced by $PK_{c}^{\prime }=\left(x_{c}^{\prime }P,R_{c}^{\prime }P\right)$. If $x_{c}^{\prime }\neq x_{c}$ (or $r_{c}^{\prime }\neq r_{c}$ ), $A_{1}$ must send $x_{c}^{\prime }$ (or $r_{c}^{\prime }$) to B. B first executes the PPKG algorithm and FSKS algorithm using identity $PID_{c}$ to obtain $SK_{c}$, and then executes the signcrypt algorithm with tuple $\left(m,ID_{u},PK_{u},PID_{c},SK_{c}\right)$ to output the ciphertext σ as $A_{1}$’s response.

(2) If $PID_{c}\notin L_{RC}$ and $PID_{c}\neq PID^{♢}$, B first executes the PPKG algorithm and FSKS algorithm using identity $PID_{c}$ to obtain $SK_{c}$, and then executes the signcrypt algorithm with tuple $\left(m,ID_{u},PK_{u},PID_{c},SK_{c}\right)$ to output the ciphertext σ as $A_{1}$’s response.

(3) If $PID_{c}\notin L_{RC}$ and $PID_{c}=PID^{♢}$, B fails and terminates the process.

$Q_{un}\left(\sigma ,ID_{u},PK_{u},PID_{c},PK_{c}\right)$: When $A_{1}$ provides tuple $\left(\sigma ,ID_{u},PK_{u},PID_{c},PK_{c}\right)$ for a query, B performs as follows:

(1) If $ID_{u}\in L_{RU}$, then $PK_{u}=\left(x_{u}P,r_{u}P\right)$ is replaced by $PK_{u}^{\prime }=\left(x_{u}^{\prime }P,r_{u}^{\prime }P\right)$. If $x_{u}^{\prime }\neq x_{u}$ (or $r_{u}^{\prime }\neq r_{u}$ ), $A_{1}$ must send $x_{u}^{\prime }$ (or $r_{u}^{\prime }$) to B. B first executes the PPKG algorithm and FSKS algorithm using identity $ID_{u}$ to obtain $SK_{u}$, and then executes the signcrypt algorithm with tuple $\left(\sigma ,ID_{u},SK_{u},PID_{c},PK_{c}\right)$ to output the plaintext *m* or ⊥ as $A_{1}$’s response.

(2) If $PID_{c}\notin L_{Ru}$, B first executes the PPKG algorithm and FSKS algorithm using identity $ID_{u}$ to obtain $SK_{u}$, and then executes the signcrypt algorithm with tuple $\left(\sigma ,ID_{u},SK_{u},PID_{c},PK_{c}\right)$ to output the plaintext *m* or ⊥ as $A_{1}$’s response.

**Forge phase**: $A_{1}$ outputs a tuple ($\sigma^{*}=\left(\theta^{*},K^{*},\omega^{*}\right),ID_{u}^{*},PK_{u}^{*},PID_{c}^{*},PK_{c}^{*}$) and wins Game 3.

**Solving CCA problem**: If $PID_{c}^{*}\neq PID^{♢}$, then B fails. Otherwise, $PID_{c}^{*}=PID^{♢}$, then $PK_{c}^{*}=PK^{♢}=\left(x^{♢}P,\alpha P\right)$. Since $\sigma^{*}$ is a valid ciphertext, it follows that $\omega^{*}=\frac{1}{d_{c}^{*}+\rho^{*}x_{c}^{*}}P$. B proceeds with the following steps:

(1) Searches for $\left(ID_{u}^{*},x_{u}^{*}P,x_{u}^{*},r_{u}^{*}P,r_{u}^{*}\right)$ in the list $L_{UU}$.

(2) Sets $PK_{u}^{*}=\left(x_{u}^{*}P,r_{u}^{*}P\right)$.

(3) Calcuates $l_{u}^{*}=H_{2}\left(ID_{u}^{*},R_{u}^{*}\right)$, $d_{u}^{*}=r_{u}^{*}+l_{u}^{*}\delta$.

(4) Searches for $\left(PID^{♢},x^{♢}P,x^{♢},\alpha P,\nabla \right)$ in the list $L_{UC}$.

(5) Sets $PK_{c}^{*}=PK^{♢}=\left(x^{♢}P,\alpha P\right)$.

(6) Calcuates $l_{c}^{*}=H_{2}\left(PID^{♢},\alpha P\right)$, $\lambda^{*}=H_{3}\left(K^{*},ID_{u}^{*},PK_{u}^{*},PID_{c}^{*},PK_{c}^{*}\right)$,

$\tau^{*}=\left(d_{u}^{*}+\lambda^{*}x_{u}^{*}\right)K^{*}$, $\rho^{*}=H_{4}\left(\theta^{*},K^{*},ID_{u}^{*},PK_{u}^{*},PID_{c}^{*},PK_{c}^{*}\right)$,

$m^{*}=H_{5}\left(K^{*},\tau^{*},ID_{u}^{*},PK_{u}^{*},PID_{c}^{*},PK_{c}^{*}\right)⊕\theta^{*}$, $\gamma =l_{c}^{*}\delta +\rho^{*}x_{c}^{*}$ (where $x_{c}^{*}=x^{♢}$).

(7) Generates $\left(\gamma ,\omega^{*}\right)$.

$$\begin{array}{cc} \left(\gamma ,\omega^{*}\right) & =(\gamma ,\frac{1}{d_{c}^{*}+\rho^{*}x_{c}^{*}}P)\\ \; & =(\gamma ,\frac{1}{\alpha +l_{c}^{*}\delta +\rho^{*}x_{c}^{*}}P)\\ \; & =(\gamma ,\frac{1}{\alpha +\gamma }P) \end{array}$$

Therefore, $\left(\gamma ,\omega^{*}\right)$ serves as the response to the CCA problem.

Probability: Let $q_{UC}$, $q_{RC}$, $q_{KC}$ and $q_{SC}$ represent the number of $A_{1}$ executes $Q_{upk}\left(PID_{c}\right)$, $R_{upk}\left(PID_{c}\right)$, $Q_{ppk}\left(PID_{c}\right)$ and $Q_{sc}\left(\sigma ,ID_{u},PK_{u},PID_{c},PK_{c}\right)$, respectively. Next, we will calculate the probability of B successfully solving a given CCA problem. To facilitate understanding, we defined the following three events:

$\pi_{1}$: $A_{1}$ has neither operated $Q_{ppk}\left(PID^{♢}\right)$ nor replaced the value of $R_{c}^{♢}\left(\alpha P\right)$.

$\pi_{2}$: $A_{1}$ has not failed in $Q_{sc}\left(\right)$.

$\pi_{3}$: $PID_{c}^{*}=PID^{♢}$.

Because if $A_{1}$ replaces the public key of $PID_{c}$, it cannot perform $Q_{ppk}\left(\right)$ for $PID_{c}$, therefore $L_{RC}\cap L_{KC}=\emptyset$. Based on the analysis, we can obtain the following results:

$$\begin{array}{cc} \; & \mathrm{Pr}\left[\pi_{1}\right]=\frac{q_{UC}-q_{RC}-q_{KC}}{q_{UC}}\\ \; & \mathrm{Pr}\left[\pi_{2}|\pi_{1}\right]={\left(1-\frac{1}{q_{UC}}\right)}^{q_{SC}}\approx e^{-\frac{q_{SC}}{q_{UC}}}\\ \; & \mathrm{Pr}\left[\pi_{3}|\pi_{1}\wedge \pi_{2}\right]=\frac{1}{q_{UC}-q_{RC}-q_{KC}} \end{array}$$

Then, the following results can be derived:

$$\begin{array}{cc} \mathrm{Pr}[B_{success}] & =\mathrm{Pr}[\pi_{1}\wedge \pi_{2}\wedge \pi_{3}]\\ \; & =\mathrm{Pr}\left[\pi_{1}\right]\mathrm{Pr}\left[\pi_{2}|\pi_{1}\right]\mathrm{Pr}\left[\pi_{3}|\pi_{1}\wedge \pi_{2}\right]\\ \; & \approx \frac{q_{UC}-q_{RC}-q_{KC}}{q_{UC}}e^{-\frac{q_{SC}}{q_{UC}}}\cdot \frac{1}{q_{UC}-q_{RC}-q_{KC}}\\ \; & =\frac{1}{q_{UC}}e^{-\frac{q_{SC}}{q_{UC}}} \end{array}$$

Consequently, if $A_{1}$ can forge a real ciphertext with advantage ε, then B can resolve the DDH problem with a probability of $\frac{\varepsilon }{q_{UC}}e^{-\frac{q_{SC}}{q_{UC}}}$. □

**Lemma 4.**  *If the CCA problem is hard, our scheme is proven to be UF-CMA against the adversary $A_{2}$ in the SM.*

**Proof.**  Given the tuple (P,αP). The goal of B is to output the tuple $\left(\gamma ,\frac{1}{\alpha +\gamma }P\right)$.

**Initialization phase:** Same as the initialization phase in Lemma 2.

**Query phase:** B sets $PID^{♢}$ as the challenge target identity. $A_{2}$ can conduct the following types of queries, and B simulates $A_{1}$’s queries as follows:

$Q_{pid}\left(ID_{c}\right)$: Similar to Lemma 1.

$Q_{upk}\left(PID_{c}\right)$: B maintains the list $L_{UC}$, which includes the tuple $\left(PID_{c},X_{c},x_{c},R_{c},r_{c}\right)$. When $A_{2}$ provides an identity $PID_{c}$ for a query, if the $PID_{c}$ is on the list $L_{UC}$, B returns $PK_{c}$ as $A_{2}$’s response. Otherwise, $PID_{c}$ is queried as a new identity, B performs the subsequent steps:

(1) If $PID_{c}=ID^{♢}$, B randomly chooses $r^{♢}\in Z_{q}^{*}$, sets $PK_{c}=PK^{♢}=\left(\alpha P,r^{♢}P\right)$ as $A_{2}$’s response, and adds the tuple $\left(PID^{♢},\alpha P,\nabla ,r^{♢}P,r^{♢}\right)$ to the list $L_{UC}$ (where ∇ represents a null value).

(2) If $PID_{c}\neq ID^{♢}$, B randomly chooses $x_{c},r_{c}\in Z_{q}^{*}$, set $PK_{c}=\left(x_{c}P,r_{c}P\right)$ as $A_{2}$’s response, and adds the tuple $\left(PID_{c},x_{c}P,x_{c},r_{c}P,r_{c}\right)$ to the list $L_{UC}$.

$Q_{upk}\left(ID_{u}\right)$: Similar to Lemma 3.

$R_{upk}\left(PID_{c},PK_{c}^{\prime }\right)$: Similar to Lemma 1.

$R_{upk}\left(ID_{u},PK_{u}^{\prime }\right)$: Similar to Lemma 1.

$Q_{ppk}\left(PID_{c}\right)$: Similar to Lemma 1.

$Q_{ppk}\left(ID_{u}\right)$: Similar to Lemma 3.

$Q_{sv}\left(PID_{c}\right)$: B maintains the list $L_{VC}$, which includes the tuple $\left(PID_{c},x_{c}\right)$. When $A_{2}$ provides an identity $PID_{c}$ for a query, B performs the subsequent steps:

(1) If $PID_{c}=PID^{♢}$, then B fails and terminates the process.

(2) If $PID_{c}\neq PID^{♢}$, B searches for $\left(PID_{c},x_{c}P,x_{c},r_{c}P,r_{c}\right)$ in the list $L_{VC}$, outputs $x_{c}$ as $A_{2}$’s response, and then adds $\left(PID_{c},x_{c}\right)$ to the list $L_{VC}$.

$Q_{sv}\left(ID_{u}\right)$: Similar to Lemma 1.

$Q_{sc}\left(m,ID_{u},PK_{u},PID_{c},PK_{c}\right)$: Similar to Lemma 3.

$Q_{un}\left(\sigma ,ID_{u},PK_{u},PID_{c},PK_{c}\right)$: Similar to Lemma 3.

**Forge phase**: $A_{2}$ outputs the tuple $\left(\sigma^{*}=\left(\theta^{*},K^{*},\omega^{*}\right),ID_{u}^{*},PK_{u}^{*},PID_{c}^{*},PK_{c}^{*}\right)$ and wins Game 4.

**Solving the CCA problem**: If $PID_{c}^{*}\neq PID^{♢}$, then B fails. Otherwise, $PID_{c}^{*}=PID^{♢}$, then $PK_{c}^{*}=PK^{♢}=\left(\alpha P,r^{♢}P\right)$. Since $\sigma^{*}$ is a valid ciphertext, it follows that $\omega^{*}=\frac{1}{d_{c}^{*}+\rho^{*}x_{c}^{*}}P$. B proceeds with the following steps:

(1) Searches for $\left(ID_{u}^{*},x_{u}^{*}P,x_{u}^{*},r_{u}^{*}P,r_{u}^{*}\right)$ in the list $L_{UU}$.

(2) Sets $PK_{u}^{*}=\left(x_{u}^{*}P,r_{u}^{*}P\right)$.

(3) Calculates $l_{u}^{*}=H_{2}\left(ID_{u}^{*},R_{u}^{*}\right)$, $d_{u}^{*}=r_{u}^{*}+l_{u}^{*}\delta$.

(4) Searches for $\left(PID^{♢},\alpha P,\Delta ,r^{♢}P,r^{♢}\right)$ in the list $L_{UC}$.

(5) Sets $PK_{c}^{*}=PK^{♢}=\left(\alpha P,r^{♢}P\right)$.

(6) Calculates $l_{c}^{*}=H_{2}\left(PID^{♢},r^{♢}P\right)$, $\lambda^{*}=H_{3}\left(K^{*},ID_{u}^{*},PK_{u}^{*},PID_{c}^{*},PK_{c}^{*}\right)$, $\tau^{*}=\left(d_{u}^{*}+\lambda^{*}x_{u}^{*}\right)K^{*}$, $\rho^{*}=H_{4}\left(\theta^{*},K^{*},ID_{u}^{*},PK_{u}^{*},PID_{c}^{*},PK_{c}^{*}\right)$, $m^{*}=H_{5}\left(K^{*},\tau^{*},ID_{u}^{*},PK_{u}^{*},PID_{c}^{*},PK_{c}^{*}\right)⊕\theta^{*}$, $\gamma ={\rho^{*}}^{-1}\left(r_{c}^{*}+l_{c}^{*}\delta \right)$ (where $r_{c}^{*}=r^{♢}$).

(7) Generates $\left(\gamma ,\rho^{*}\omega^{*}\right)$.

$$\begin{array}{cc} \left(\gamma ,\rho^{*}\omega^{*}\right) & =(\gamma ,\rho^{*}\frac{1}{d_{c}^{*}+\rho^{*}x_{c}^{*}}P)\\ \; & =(\gamma ,\rho^{*}\frac{1}{r_{c}^{*}+l_{c}^{*}\delta +\alpha \rho^{*}}P)\\ \; & =(\gamma ,\frac{1}{{\rho^{*}}^{-1}\left(r_{c}^{*}+l_{c}^{*}\delta \right)+\alpha }P)\\ \; & =(\gamma ,\frac{1}{\alpha +\gamma }P) \end{array}$$

Therefore, $\left(\gamma ,\omega^{*}\right)$ serves as the response to the CCA problem.

Probability: Let $q_{UC}$, $q_{RC}$, $q_{VC}$ and $q_{SC}$ represent the number of $A_{2}$ executing $Q_{upk}\left(PID_{c}\right)$, $R_{upk}\left(PID_{c}\right)$, $Q_{sv}\left(PID_{c}\right)$ and $Q_{sc}\left(\sigma ,ID_{u},PK_{u},PID_{c},PK_{c}\right)$, respectively. Next, we will calculate the probability of B successfully solving a given CCA problem. To facilitate understanding, we defined the following three events:

$\pi_{1}$: $A_{2}$ has neither operated $Q_{sv}\left(PID^{♢}\right)$ nor replaced the value of $X_{c}^{♢}\left(\alpha P\right)$.

$\pi_{2}$: $A_{2}$ has not failed in $Q_{sc}\left(\right)$.

$\pi_{3}$: $PID_{c}^{*}=PID^{♢}$.

Because if $A_{2}$ replaces the public key of $PID_{c}$, it cannot perform $Q_{sv}\left(\right)$ for $PID_{c}$, therefore $L_{RC}\cap L_{VC}=\emptyset$. Based on the analysis, we can obtain the following results:

$$\begin{array}{cc} \; & \mathrm{Pr}\left[\pi_{1}\right]=\frac{q_{UC}-q_{RC}-q_{VC}}{q_{UC}}\\ \; & \mathrm{Pr}\left[\pi_{2}|\pi_{1}\right]={\left(1-\frac{1}{q_{UC}}\right)}^{q_{SC}}\approx e^{-\frac{q_{SC}}{q_{UC}}}\\ \; & \mathrm{Pr}\left[\pi_{3}|\pi_{1}\wedge \pi_{2}\right]=\frac{1}{q_{UC}-q_{RC}-q_{VC}} \end{array}$$

Then, the following results can be derived:

$$\begin{array}{cc} \mathrm{Pr}[B_{success}] & =\mathrm{Pr}[\pi_{1}\wedge \pi_{2}\wedge \pi_{3}]\\ \; & =\mathrm{Pr}\left[\pi_{1}\right]\mathrm{Pr}\left[\pi_{2}|\pi_{1}\right]\mathrm{Pr}\left[\pi_{3}|\pi_{1}\wedge \pi_{2}\right]\\ \; & \approx \frac{q_{UC}-q_{RC}-q_{VC}}{q_{UC}}e^{-\frac{q_{SC}}{q_{UC}}}\cdot \frac{1}{q_{UC}-q_{RC}-q_{VC}}\\ \; & \approx \frac{1}{q_{UC}}e^{-\frac{q_{SC}}{q_{UC}}} \end{array}$$

Consequently, if $A_{2}$ can forge a real ciphertext with advantage ε, then B can resolve the DDH problem with a probability of $\frac{\varepsilon }{q_{UC}}e^{-\frac{q_{SC}}{q_{UC}}}$. □

**Theorem 2.**  *If the CCA problem is hard, our scheme is proven to be UF-CMA in the SM.*

**Proof.**  From Lemmas 3 and 4, we can see that the conclusion is correct. □

**Theorem 3.**  *If the DDH problem is hard, our scheme is proven to be anonymous to the signcrypter against the super adversary A in the SM.*

**Proof.**  Given the tuple (P,αP,βP,T), where $\alpha ,\beta \in Z_{q}^{*}$ and α,β are unknown. The goal of B is to determine whether *T* is equal to aβP.

**Initialization phase:** Same as the initialization phase in Lemma 2.

**Query phase:** A inputs various queries, and B executes the corresponding algorithm to generate the answer.

**Challenge phase:** A selects a message $m^{*}$ and two distinct real identities $ID_{0}^{*}$ and $ID_{1}^{*}$ of C, where A has not performed $Q_{pid}$ for $ID_{0}^{*}$ and $ID_{1}^{*}$. A subsequently sends tuple $\left(m^{*},ID_{0}^{*},ID_{1}^{*},ID_{u}^{*},PK_{u}^{*}\right)$ to B. B performs the subsequent steps:

(1) Randomly chooses ξ∈{0,1}.

(2) Sets $E_{\xi }^{*}=\alpha P,F_{\xi }^{*}=\beta P$.

(3) Calculates $PID_{\xi }^{*}=ID_{\xi }^{*}⊕H_{1}\left(\delta T,E_{\xi }^{*},F_{\xi }^{*}\right)$

(4) Runs the PPKG algorithm, SVS algorithm and FSKS algorithm to acquire the private key $SK_{\xi }^{*}$ of $PID_{\xi }^{*}$.

(5) Runs the signcrypt algorithm on the tuple $\left(m^{*},ID_{u}^{*},PK_{u}^{*},PID_{\xi }^{*},SK_{\xi }^{*}\right)$ to acquire the ciphertext $\sigma^{*}$.

(6) Outputs $\left(\sigma^{*},ID_{u}^{*},PK_{u}^{*},PID_{\xi }^{*},PK_{\xi }^{*}\right)$ to A.

**Guess phase**: A performs various queries adaptively, as in the query phase. After that, A outputs its guess $\xi^{\prime }\in \left\{0,1\right\}$.

**Solving the DDH problem**: B returns “1”, if $\xi^{\prime }=\xi$. Otherwise, B outputs “0”. If T=αβP, then

$$\begin{array}{cc} PID_{\xi }^{*} & =ID_{\xi }^{*}⊕H_{1}\left(\delta T,E_{\xi }^{*},F_{\xi }^{*}\right)\\ \; & =ID_{\xi }^{*}⊕H_{1}\left(\delta \alpha \beta P,\alpha P,\beta P\right) \end{array}$$

This means that $PID_{\xi }^{*}$ is a true pseudo-identity. Therefore, the advantage of A in distinguishing symbol ξ is ε, that is to say:

$$\mathrm{Pr}\left[B\to 1|T=\alpha \beta P\right]=\mathrm{Pr}\left[\xi^{\prime }=\xi |T=\alpha \beta P\right]=\frac{1}{2}+\varepsilon .$$

If T≠αβP, then $PID_{\xi }^{*}$ is not a true pseudo-identity. This implies that for this $\sigma^{*}$, the distribution of ξ=0 and ξ=1 is the same. Therefore, A cannot have any advantage in identifying symbol ξ, that is to say:

$$\mathrm{Pr}\left[B\to 1|T\neq \alpha \beta P\right]=\mathrm{Pr}\left[\xi^{\prime }=\xi |T\neq \alpha \beta P\right]=\frac{1}{2}.$$

During the proof process, C will not fail.Consequently, if A can distinguish symbol ξ with the advantage ε, then B can resolve the DDH problem with a probability of ε. □

**Theorem 4.**  *Our scheme is identifiable.*

**Proof.**  Proof: The KGC can generate the $params=\{G_{1},G_{2},q,e,P,P_{pub}=\delta P,N=e\left(P,P\right),$$H_{1}∼H_{5}\}$ and the msk={δ}. Let $\left(\sigma =\left(\theta ,K,\omega \right),ID_{u},PK_{u},PID_{c},PK_{c}\right)$ be a legitimate ciphertext. The KGC then performs the subsequent steps:

(1) Searches for $\left(PID_{c},e_{c},e_{c}P,f_{c},f_{c}P\right)$ in the list FI.

(2) Computes $\Delta C=\delta e_{c}f_{c}P$, $ID_{c}=PID_{c}⊕H_{1}\left(\Delta C,e_{c}P,f_{c}P\right)$.

(3) Outputs the true identity $ID_{c}$ of C.

Thus, for any ciphertext, the KGC can identify the true identity of C. So our scheme is identifiable. □

## 7. Performance Analysis

In this section, we delve into a comprehensive evaluation of our scheme’s security properties, functionalities, computational expenses, and storage costs. Additionally, we compare its performance with the schemes presented in [28,30,32,40,46,49,50,51].

### 7.1. Security Analysis

Firstly, we analyze the security properties and functionalities of our scheme. Theorem 1 indicates that adversaries are unable to obtain valid messages, thus ensuring that our scheme can achieve confidentiality. Theorem 2 demonstrates that no adversary can forge legitimate signatures. Therefore, our scheme can simultaneously satisfy confidentiality, integrity, authentication, and non-repudiation. Theorem 3 indicates that our scheme provides anonymity for the signcryptor. Theorem 4 demonstrates that our scheme is also identity identifiable. Furthermore, our scheme is characterized by public verifiability, public ciphertext authenticity, and is classed as certificateless cryptography.

Secondly, compare the security properties and functional of our scheme with those of the schemes in [28,30,32,40,46,49,50,51]. The comparison results are shown in Table 1, where SM represents the standard model, ROM represents the random oracle model, √ represents the scheme compliance attribute, × represents the scheme non-compliance attribute, and - represents unknown. As shown in Table 1, our scheme satisfies the four security properties of confidentiality, integrity, authentication, non-repudiation. These properties have been proven within the standard model. Since our security model has been enhanced, our scheme stands out as the most secure among all schemes. Furthermore, compared to other schemes, only our scheme concurrently realizes all four functions: anonymity of the signcryptor, identity identifiability, public verifiability, and public ciphertext authenticity, while incorporating a certificateless design. Notably, the anonymity of the signcryptor and identity identifiability can be proven in the standard model. Therefore, our scheme is not only more secure but also has more comprehensive functionalities.

### 7.2. Efficiency Analysis

Moving forward, we proceed to compare the computational expenses associated with the previously discussed schemes. To facilitate the comparison, we adopt the computation time of the scheme by He et al. [52] as the benchmark. The relevant operations were implemented using the well-known cryptographic library (MIRACL) on a smartphone (Samsung Galaxy S5 G9001, Qualcomm Snapdragon 801 Quad-core 2.5 GHz Krait 400, GPU Adreno 330, 16GB 2GB RAM, Android 4.4.2 KitKat, Samsung Electronics, Seoul, Republic of Korea). The symbols for various operations and their precise running times are detailed in Table 2. The function $e:G_{1}\times G_{1}\to G_{2}$ is defined as a bilinear pairing. In this context, $G_{1}$ represents an additive group of prime order *q*, which is constructed on the basis of a singular elliptic curve group over a finite field $F_{p}$ of prime order. The bit size associated with *p* and *q* are designated as 512 and 160 bits, respectively.

**Table 1:** Comparison of the security properties and functionalities.

Schemes	Confidentiality	Integrity	Authentication	Non-Repudiation	Anonymity of the Signcrypter	Identity Identifiability	Public Verifiability	Public Ciphertext Authenticity	Certificateless	Security Model
Luo [28]	×	×	×	×	×	×	×	×	√	SM
Rastegari [40]	×	√	√	√	×	×	×	×	√	SM
Zhou [30]	√	√	√	√	×	×	×	×	√	SM
Zhou [53]	√	√	√	√	×	×	×	×	√	SM
Lu [46]	-	√	√	√	×	×	×	×	×	ROM
Deng [49]	√	√	√	√	√	√	×	×	√	SM
Li [32]	√	√	√	√	×	×	×	×	√	ROM
Luo [51]	√	√	√	√	×	×	√	√	√	ROM
Karati [50]	√	√	√	√	×	×	√	√	√	SM
our	√	√	√	√	√	√	√	√	√	SM

In terms of the computational efficiency of the CLSC scheme, its performance mainly depends on the computational costs of the signcryption and unsigncryption algorithms. For this reason, we focus solely on the computational costs of these two algorithms. To compare computational complexity more effectively, our primary focus lies in comparing the two most time-consuming operations: bilinear pairing operation and hash-to-point operation, so as to more accurately evaluate the performance differences between them. From Table 3, we observe that in the signcryption algorithm, our scheme requires only one bilinear pairing operation and does not necessitate any hash-to-point operations. In contrast, scheme [53] necessitates up to five bilinear pairing operations, while scheme [46] requires one bilinear pairing operation in addition to two hash-to-point operations. Although schemes [32,49,50,51] employ fewer instances of both operations compared to ours, our overall time consumption remains lower than theirs. In the unsigncryption algorithm, our scheme requires only one bilinear pairing operation and does not necessitate any hash-to-point operations. Other schemes perform bilinear pairing operation at least twice, but do not involve hash-to-point operation. Overall, with the exception of scheme [50] our scheme provably employs a lower number of bilinear pairing operations and hash-to-point operations compared to all other schemes. Moreover, our total time consumption is still lower than all other schemes. Therefore, our scheme boasts the lowest computational complexity. Below is a detailed analysis.

**Figure 3:**
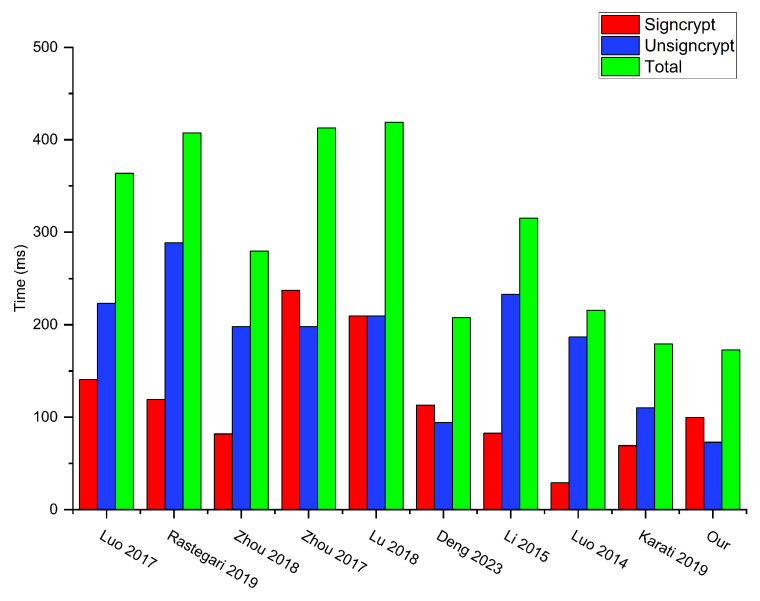
Comparison of computational overheads [28,30,32,40,46,49,50,51,53].

We measured the computational overheads for the various schemes, as illustrated in Table 3 and Figure 3. According to the scheme [28], the computational overhead of the signcryption algorithm, unsigncryption algorithm, and the total are $3T_{sm_{G_{1}}}+T_{exp_{G_{2}}}+3T_{bp}$, $2T_{sm_{G_{1}}}+6T_{bp}$, and $5T_{sm_{G_{1}}}+T_{exp_{G_{2}}}+9T_{bp}=363.691$ ms, respectively. In the scheme [40], the computational overhead of the signcryption algorithm, unsigncryption algorithm, and total are $4T_{sm_{G_{1}}}+2T_{bp}$, $2T_{sm_{G_{1}}}+8T_{bp}$, $6T_{sm_{G_{1}}}+10T_{bp}=407.56$ ms, respectively. In the scheme [30], the computational overhead of the signcryption algorithm, unsigncryption algorithm, and the total are $3T_{sm_{G_{1}}}+4T_{exp_{G_{2}}}+T_{bp}$, $5T_{sm_{G_{1}}}+4T_{bp}$, and $8T_{sm_{G_{1}}}+4T_{exp_{G_{2}}}+5T_{bp}=279.801$ ms, respectively. In the scheme [53], the computational overhead of the signcryption algorithm, unsigncryption algorithm, and the total are $5T_{sm_{G_{1}}}+3T_{exp_{G_{2}}}+5Tbp$, $3T_{sm_{G_{1}}}+2T_{exp_{G_{2}}}+4T_{bp}$, and $8T_{sm_{G_{1}}}+5T_{exp_{G_{2}}}+9T_{bp}=412.902$ ms, respectively. In the scheme [46], the computational overhead of the signcryption algorithm, unsigncryption algorithm, and the total are $8T_{sm_{G_{1}}}+T_{exp_{G_{2}}}+T_{bp}+2T_{htp}$, $T_{sm_{G_{1}}}+6T_{bp}$, and $9T_{sm_{G_{1}}}+T_{exp_{G_{2}}}+7T_{bp}+2T_{htp}=419.049$ ms, respectively. In the scheme [49], the computational overhead of the signcryption algorithm, unsigncryption algorithm, and the total are $6T_{sm_{G_{1}}}+T_{bp}$, $2T_{sm_{G_{1}}}+T_{exp_{G_{2}}}+2T_{bp}$, and $8T_{sm_{G_{1}}}+T_{exp_{G_{2}}}+3T_{bp}=207.628$ ms, respectively. In the scheme [32], the computational overhead of the signcryption algorithm, unsigncryption algorithm, and the total are $6T_{sm_{G_{1}}}+T_{exp_{G_{2}}}$, $5T_{sm_{G_{1}}}+T_{exp_{G_{2}}}+5T_{bp}$, and $11T_{sm_{G_{1}}}+2T_{exp_{G_{2}}}+5T_{bp}=315.518$ ms, respectively. In the scheme [51], the computational overhead of the signcryption algorithm, unsigncryption algorithm, and the total are $2T_{sm_{G_{1}}}+T_{exp_{G_{2}}}$, $4T_{sm_{G_{1}}}+T_{exp_{G_{2}}}+4T_{bp}$, and $6T_{sm_{G_{1}}}+2T_{exp_{G_{2}}}+4T_{bp}=215.78$ ms, respectively. In the scheme [50], the computational overhead of the signcryption algorithm, unsigncryption algorithm, and the total are $5T_{sm_{G_{1}}}+T_{exp_{G_{2}}}$, $3T_{sm_{G_{1}}}+2T_{exp_{G_{2}}}+2T_{bp}$, and $8T_{sm_{G_{1}}}+3T_{exp_{G_{2}}}+2T_{bp}=179.413$ ms, respectively. In our scheme, the computational overhead of the signcryption algorithm, unsigncryption algorithm, and the total are $5T_{sm_{G_{1}}}+T_{bp}=99.738$, $3T_{sm_{G_{1}}}+T_{bp}=72.928$, and $8T_{sm_{G_{1}}}+2T_{bp}=172.666$ ms, respectively.

Based on Figure 3 and the analysis above, the computational cost for unsigncryption in our scheme is lower than all other schemes. While the schemes [30,32,50,51] have a lower computational cost for signcryption than ours, they suffer from a lack of critical functionalities. Specifically, schemes [30,32] do not provide anonymity of the signcrypter, identity identifiability, public verifiability, and public ciphertext authenticity. Additionally, schemes [50,51] also lack anonymity of the signcrypter and identity identifiability. In contrast, our scheme maintains a balance between computational efficiency, the essential security and critical functionalities. In terms of total cost, the total cost of our scheme is the lowest, and it can be observed that the total computational overhead for our scheme is approximately 47.48% of the scheme [28], 42.37% of the scheme [40], 61.71% of the scheme [30], 41.82% of the scheme [53], 41.20% of the scheme [46], 83.16% of the scheme [49], 54.72% of the scheme [32], 80.02% of the scheme [51], and 96.24% of the scheme [50].

**Figure 4:**
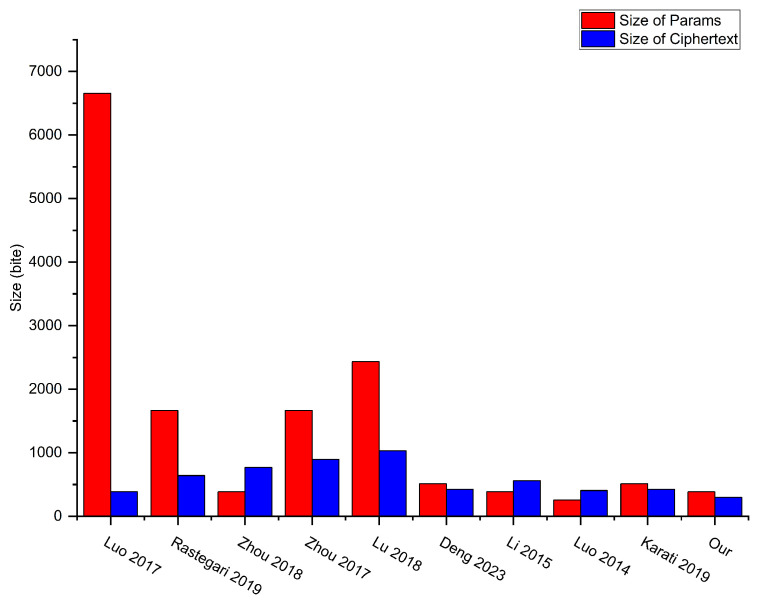
Comparison of storage costs [28,30,32,40,46,49,50,51,53].

Next, we compare the storage costs of the schemes, as shown in Table 4 and Figure 4. Let $|G_{1}|$, $|G_{2}|$, $|Z_{q}^{*}|$ denote the size of elements in $G_{1},G_{2}$ and $Z_{q}^{*}$, respectively. Accordingly, we have $|G_{1}\left|=\right|G_{2}|=128$ bytes, and $|Z_{q}^{*}|=20$ bytes. The size of the output produced by the hash function is denoted as η=40 bytes, the output size of the identity information is denoted as δ=8 bytes, and the attribute size is denoted as τ. Let us assume τ=δ=8 bytes.

The size of the system parameters in the scheme [28,30,32,40,46,49,50,51,53], and our scheme are $\left(\delta +\eta +4\right)|G_{1}|=\left(8+40+4\right)\times 128=6656$ bytes, $\left(\delta +4\right)|G_{1}\left|+\right|G_{2}|=\left(8+4\right)\times 128+128=1664$ bytes, $3|G_{1}|=3\times 128=384$ bytes, $\left(\delta +5\right)|G_{1}|=\left(8+5\right)\times 128=1664$ bytes, $\left(2+\delta +\tau \right)|G_{1}\left|+\right|G_{2}|=\left(2+8+8\right)\times 128+128=2432$ bytes, $3|G_{1}\left|+\right|G_{2}|=3\times 128+128=512$ bytes, $|G_{1}\left|+2\right|G_{2}|=128+2\times 128=384$ bytes, $2|G_{1}|=2\times 128=256$ bytes, $3|G_{1}\left|+\right|G_{2}|=3\times 128+128=512$ bytes, and $2|G_{1}\left|+\right|G_{2}|=2\times 128+128=$ 384 bytes, respectively.

The length of the ciphertext in the scheme [28,30,32,40,46,49,50,51,53], and our scheme are $2|G_{1}\left|+\right|G_{2}|=2\times 128+128=384$ bytes, $4|G_{1}\left|+\right|G_{2}|=4\times 128+128=640$ bytes, $3|G_{1}\left|+3\right|G_{2}|=3\times 128+3\times 128=768$ bytes, $4|G_{1}\left|+3\right|G_{2}|=4\times 128+3\times 128=896$ bytes, $7|G_{1}\left|+\right|G_{2}|+\delta =7\times 128+128+8=1032$ bytes, $3|G_{1}|+\eta =3\times 128+40=424$ bytes, $4|G_{1}\left|+2\right|Z_{q}^{*}|+\delta =4\times 128+2\times 20+8=560$ bytes, $2|G_{1}\left|+\right|G_{2}\left|+\right|Z_{q}^{*}|=2\times 128+128+20=404$ bytes, $2|G_{1}\left|+\right|G_{2}|+\eta =2\times 128+128+40=424$ bytes, and $2|G_{1}|+\delta =2\times 128+8=296$ bytes, respectively.

Based on Figure 4 and the previous detailed analysis, our scheme exhibits a notable advantage in ciphertext length, surpassing all other schemes except for scheme [51]. Nevertheless, it must be pointed out that while scheme [51] is relatively close to us in ciphertext length, it fails to offer the two crucial features of anonymity of the signcrypter and identity identifiability. Furthermore, the security proof of scheme [51] relies on the random oracle model, which to some extent undermines its universality and reliability in practical applications. Notably, in terms of the length of system parameters, our scheme achieves the shortest length, which fully demonstrates its superiority in efficiency. It can be observed that the system parameter size of our scheme is approximately 5.77% of the scheme [28], 23.08% of the scheme [40], 100% of the scheme [30], 23.08% of the scheme [53], 15.79% of the scheme [46], 60.76% of the scheme [49], 100% of the scheme [32], 150% of the scheme [51], and 75% of the scheme [50]. The ciphertext size of our scheme is approximately 77.08% of the scheme [28], 46.25% of the scheme [40], 38.54% of the scheme [30], 33.04% of the scheme [53], 28.66% of the scheme [46], 69.81% of the scheme [49], 52.86% of the scheme [32], 73.27% of the scheme [51], and 69.81% of the scheme [50].

In conclusion, our CL-ASC scheme has demonstrated all crucial security properties in the standard model, and it is also more comprehensive in terms of functionality, particularly in offering anonymity of the signcrypter and identity identifiability. With the exception of the scheme [51], which holds a slight advantage in terms of system parameter size, our scheme outperforms all other known schemes in both computational overhead and storage costs. Consequently, compared to existing schemes, our CL-ASC scheme boasts lower computational and storage costs while maintaining a higher level of security. This makes it an ideal and cost-effective communication solution for WBANs.

The ethical and regulatory issues surrounding signcryption schemes primarily manifest in the following aspects: Technical Ethics: Signcryption schemes require ensuring that the technology, in its design and implementation, is not only secure and reliable but also respects user privacy, is transparent and auditable, and adheres to ethical and legal standards.This includes utilizing robust encryption algorithms to safeguard data, adopting decentralized storage to mitigate privacy risks, implementing data minimization principles to reduce the likelihood of breaches, and ensuring transparency to build trust. Social Ethics: Signcryption schemes, which can be employed to protect the communication and transactions of individuals or organizations, must be designed with social interests and public safety in mind. For instance, it should not be permissible for encryption technology to be utilized in support of illegal activities or to evade legal oversight. Individual Ethics: In the design of signcryption schemes, it is imperative to respect and protect the personal privacy of users. This implies that the processes of generating, storing, and utilizing encryption keys must ensure the confidentiality and privacy of user data. Transparency and Accountability: The provider of the signcryption scheme should clarify its responsibilities in data protection and transparently explain its data processing and protection measures to users and interested parties. Legal and Regulatory Compliance: Signcryption schemes must adhere to relevant legal and regulatory requirements, including data protection laws, electronic communication laws, and other pertinent regulations. User Education and Awareness: Users of signcryption technology should be educated about their rights and responsibilities, including how to safely use encryption tools and protect their keys. In summary, the ethical and regulatory issues surrounding signcryption schemes encompass various aspects, such as privacy protection, transparency and accountability, legal and regulatory compliance, and technological neutrality and balance, as well as user education and awareness enhancement. Addressing these issues necessitates concerted efforts and collaboration among technical designers, providers, users, and regulatory bodies.

## 8. Conclusions

Designing a secure and economical communication scheme specifically for Wireless Body Area Networks (WBANs) is a critical issue that needs urgent attention. Signcryption technology has emerged as an ideal choice for WBAN due to its ability to simultaneously achieve confidentiality, authentication, integrity, and non-repudiation at a relatively low cost. However, while the recently proposed CLSC schemes possess their own advantages, they also suffer from several drawbacks, including reliance on the ROM for security proofs, lack of public verifiability, public ciphertext authenticity and anonymity, and high computational costs. To address these issues, this paper first introduces a novel CL-ASC scheme. Second, it establish an enhanced security model for the CL-ASC scheme. Furthermore, it proves that our CL-ASC scheme possesses indistinguishability, unforgeability, and anonymity of the signcrypter within the standard model. Finally, a comparative analysis of the performance of several CLSC schemes reveals that our CL-ASC scheme has lower computational and storage costs and superior security. Consequently, our CL-ASC scheme offers a more ideal and economical communication solution tailored for WBAN applications.

## Figures and Tables

**Table 2:** Symbols.

Symbols	Definition
$T_{bp}$	The overhead it takes to execute a bilinear pairing operation, $T_{bp}\approx$ 32.713 ms
$T_{htp}$	The overhead it takes to execute a hash-to-point operation, $T_{htp}\approx$ 33.582 ms
$T_{sm_{G_{1}}}$	The overhead it takes to execute a scalar multiplication operation in $G_{1}$, $T_{sm_{G_{1}}}\approx 13.405$ ms
$T_{exp_{G_{2}}}$	The overhead it takes to execute a exponentiation operation in $G_{2}$, $T_{exp_{G_{2}}}\approx 2.249$ ms

**Table 3:** Comparison of computational overheads.

Schemes	Signcryption (ms)	Unsigncryption (ms)	Total (ms)
Luo [28]	$3T_{sm_{G_{1}}}+T_{exp_{G_{2}}}+3T_{bp}$	$2T_{sm_{G_{1}}}+6T_{bp}$	363.691
Rastegari [40]	$4T_{sm_{G_{1}}}+2T_{bp}$	$2T_{sm_{G_{1}}}+8T_{bp}$	407.56
Zhou [30]	$3T_{sm_{G_{1}}}+4T_{exp_{G_{2}}}+T_{bp}$	$5T_{sm_{G_{1}}}+4T_{bp}$	279.801
Zhou [53]	$5T_{sm_{G_{1}}}+3T_{exp_{G_{2}}}+5T_{bp}$	$3T_{sm_{G_{1}}}+2T_{exp_{G_{2}}}+4T_{bp}$	412.902
Lu [46]	$8T_{sm_{G_{1}}}+T_{exp_{G_{2}}}+T_{bp}+2T_{htp}$	$T_{sm_{G_{1}}}+6T_{bp}$	419.049
Deng [49]	$6T_{sm_{G_{1}}}+T_{bp}$	$2T_{sm_{G_{1}}}+T_{exp_{G_{2}}}+2T_{bp}$	207.628
Li [32]	$6T_{sm_{G_{1}}}+T_{exp_{G_{2}}}$	$5T_{sm_{G_{1}}}+T_{exp_{G_{2}}}+5T_{bp}$	315.518
Luo [51]	$2T_{sm_{G_{1}}}+T_{exp_{G_{2}}}$	$4T_{sm_{G_{1}}}+T_{exp_{G_{2}}}+4T_{bp}$	215.78
Karati [50]	$5T_{sm_{G_{1}}}+T_{exp_{G_{2}}}$	$3T_{sm_{G_{1}}}+2T_{exp_{G_{2}}}+2T_{bp}$	179.413
our	$5T_{sm_{G_{1}}}+T_{bp}$	$3T_{sm_{G_{1}}}+T_{bp}$	172.666

**Table 4:** Comparison of storage costs.

Scheme	Size of System Parameters (bytes)	Size of Ciphertext (bytes)
Luo [28]	$\left(\delta +\eta +4\right)|G_{1}|$ /(6656)	$2|G_{1}\left|+\right|G_{2}|$ /(384)
Rastegari [40]	$\left(\delta +4\right)|G_{1}\left|+\right|G_{2}|$/(1664)	$4|G_{1}\left|+\right|G_{2}|$ /(640)
Zhou [30]	$3|G_{1}|$/(384)	$3|G_{1}\left|+3\right|G_{2}|$/(768)
Zhou [53]	$\left(\delta +5\right)|G_{1}|$/(1664)	$4|G_{1}\left|+3\right|G_{2}|$/(896)
Lu [46]	$\left(2+\delta +\tau \right)|G_{1}\left|+\right|G_{2}|$/(2432)	$7|G_{1}\left|+\right|G_{2}|+\delta$/(1032)
Deng [49]	$3|G_{1}\left|+\right|G_{2}|$/(512)	$3|G_{1}|+\eta$/(424)
Li [32]	$|G_{1}\left|+2\right|G_{2}|$/(384)	$4|G_{1}\left|+2\right|Z_{q}^{*}|+\delta$/(560)
Luo [51]	$2|G_{1}|$/(256)	$2|G_{1}\left|+\right|G_{2}\left|+\right|Z_{q}^{*}|$ /(404)
Karati [50]	$3|G_{1}\left|+\right|G_{2}|$/(512)	$2|G_{1}\left|+\right|G_{2}|+\eta$ /(424)
our	$2|G_{1}\left|+\right|G_{2}|$/(384)	$2|G_{1}|+\delta$ /(296)

## Data Availability

Data are contained within the article.
